# Reconstituting gut microbiota-colonocyte interactions reverses diet-induced cognitive deficits: The beneficial of *eucommiae cortex* polysaccharides

**DOI:** 10.7150/thno.99468

**Published:** 2024-08-01

**Authors:** Mengli Wang, Penghao Sun, Xuejun Chai, Yong-Xin Liu, Luqi Li, Wei Zheng, Shulin Chen, Xiaoyan Zhu, Shanting Zhao

**Affiliations:** 1 College of Veterinary Medicine, Northwest A&F University, Yangling, Shaanxi 712100, China.; 2 College of Basic Medicine, Xi'an Medical University, Xi'an, Shaanxi 710000, China.; 3 Shenzhen Branch, Guangdong Laboratory of Lingnan Modern Agriculture, Genome Analysis Laboratory of the Ministry of Agriculture and Rural Affairs, Agricultural Genomics Institute at Shenzhen, Chinese Academy of Agricultural Sciences, Shenzhen, Guangdong 518120, China.; 4 Life Science Research Core Services, Northwest A&F University, Yangling, Shaanxi 712100, China.; 5 College of Resources and Environment Sciences, Northwest A&F University, Yangling, Shaanxi 712100, China.

**Keywords:** *Eucommiae cortex* polysaccharides, high-fat diet, cognitive deficits, gut microbiota, colonocyte metabolism

## Abstract

**Rationale:** Consumption of a high-fat diet (HFD) has been implicated in cognitive deficits and gastrointestinal dysfunction in humans, with the gut microbiota emerging as a pivotal mediator of these diet-associated pathologies. The introduction of plant-based polysaccharides into the diet as a therapeutic strategy to alleviate such conditions is gaining attention. Nevertheless, the mechanistic paradigm by which polysaccharides modulate the gut microbiota remains largely undefined. This study investigated the mechanisms of action of *Eucommiae cortex* polysaccharides (EPs) in mitigating gut dysbiosis and examined their contribution to rectifying diet-related cognitive decline.

**Methods:** Initially, we employed fecal microbiota transplantation (FMT) and gut microbiota depletion to verify the causative role of changes in the gut microbiota induced by HFD in synapse engulfment-dependent cognitive impairments. Subsequently, colonization of the gut of chow-fed mice with *Escherichia coli* (*E. coli*) from HFD mice confirmed that inhibition of *Proteobacteria* by EPs was a necessary prerequisite for alleviating HFD-induced cognitive impairments. Finally, supplementation of HFD mice with butyrate and treatment of EPs mice with GW9662 demonstrated that EPs inhibited the expansion of *Proteobacteria* in the colon of HFD mice by reshaping the interactions between the gut microbiota and colonocytes.

**Results:** Findings from FMT and antibiotic treatments demonstrated that HFD-induced cognitive impairments pertaining to neuronal spine loss were contingent on gut microbial composition. Association analysis revealed strong associations between bacterial taxa belonging to the phylum *Proteobacteria* and cognitive performance in mice. Further, introducing *E. coli* from HFD-fed mice into standard diet-fed mice underscored the integral role of *Proteobacteria* proliferation in triggering excessive synaptic engulfment-related cognitive deficits in HFD mice. Crucially, EPs effectively counteracted the bloom of *Proteobacteria* and subsequent neuroinflammatory responses mediated by microglia, essential for cognitive improvement in HFD-fed mice. Mechanistic insights revealed that EPs promoted the production of bacteria-derived butyrate, thereby ameliorating HFD-induced colonic mitochondrial dysfunction and reshaping colonocyte metabolism. This adjustment curtailed the availability of growth substrates for facultative anaerobes, which in turn limited the uncontrolled expansion of *Proteobacteria*.

**Conclusions:** Our study elucidates that colonocyte metabolic disturbances, which promote *Proteobacteria* overgrowth, are a likely cause of HFD-induced cognitive deficits. Furthermore, dietary supplementation with EPs can rectify behavioral dysfunctions associated with HFD by modifying gut microbiota-colonocyte interactions. These insights contribute to the broader understanding of the modulatory effects of plant prebiotics on the microbiota-gut-brain axis and suggest a potential therapeutic avenue for diet-associated cognitive dysfunction.

## Introduction

Consumption of a high-fat diet (HFD) and the resulting obesity can impose numerous stressors on the individual, including cognitive and emotional dysfunction 1. At the same time, a sustained HFD usually leads to gut dysbiosis and systemic low-grade inflammation 2. In addition, animal model studies have shown that standard diet-fed mice that received a gut microbiota transplant from donors with HFD develop neurologic dysfunction 3. These findings suggest that gut microbiota dysbiosis plays a pivotal role in the cognitive dysfunction induced by HFD. Although the link between diet-induced gut dysbiosis and individual brain dysfunction is well established, further evidence is required to elucidate the precise mechanisms by which alterations in gut bacteria in response to dietary factors regulate brain functional homeostasis. Microglia, the resident immune cells in the central nervous system (CNS), have been shown to respond to systemic low-grade inflammation and affect brain function 4, 5. Synapse loss is a notable feature of cognitive dysfunction. Therefore, increased immunological activity, followed by heightened microglial activation and excessive synaptic pruning, plays a crucial role in the spatial learning deficits 6. However, it remains to be explored whether microglia-mediated synaptic engulfment contributes to gut dysbiosis-associated cognitive impairments in HFD mice.

A fundamental mechanism involved in the development of systemic inflammation induced by gut microbiota is the release of lipopolysaccharide (LPS) produced by Gram-negative bacteria into the peripheral circulation 7. In individuals with obesity and related metabolic disorders, there is an uncontrolled increase in the abundance of LPS-producing bacteria, particularly *Proteobacteria*, in the gut 8, 9. Among gut bacteria, an overgrowth of *Proteobacteria* consistently corresponds to the ecological changes during gut dysbiosis 10. HFD feeding favorably increases the abundance of *Proteobacteria*, damaging the intestinal epithelial barrier 11, 12. As a result, dysfunction of the intestinal barrier exposes immune cells to excessive amounts of LPS and bacteria, triggering systemic inflammation and subsequent neuroinflammation. Polysaccharides play a vital role in maintaining gut ecology by regulating macronutrients and host physiology 13. One common benefit of polysaccharides from different sources is the promotion of the proliferation of beneficial bacteria while inhibiting the growth of pathogens in the intestinal tract 14, 15. This further alleviates the various diseases caused by microbiota dysbiosis. Although the beneficial effects of polysaccharides have been extensively documented, the underlying mechanisms by which they influence the composition of the gut microbiota remain poorly understood. Our study has demonstrated that *Eucommiae cortex* polysaccharides (EPs) possess the potential to alter the gut bacterial structure and exert neuroprotective effects 16. The present study further investigates the mechanisms by which EPs inhibit the expansion of pathogenic bacteria populations in the gut and seeks to determine the causal role of this outcome in ameliorating diet-related brain dysfunction.

Functional homeostasis in the colon depends on an efficient epithelial barrier and coordinated colonocyte metabolism, both of which require the support of mitochondrial function 17. Energy acquisition of colonocytes predominantly relies on the metabolism of short-chain fatty acids (SCFAs), including propionate, acetate, and butyrate, via β-oxidation 18-20. Peroxisome proliferator-activated receptor γ (PPAR-γ) signaling in colonic tissue, activated by microbiota-derived butyrate, enhances the mitochondrial activity and the β-oxidation of fatty acids, which is a highly oxygen-consuming process 18, 21. Consequently, this supports an anaerobic environment in the colonic lumen, driving the dominance of beneficial obligate anaerobic bacteria in the gut microbiota that can convert indigestible polysaccharides into SCFAs through the secretion of a diverse range of polysaccharide-degrading enzymes under anaerobic conditions 10, 22. Conversely, impaired mitochondrial activity triggers colonocyte reprogramming, leading to increased bioavailability of oxygen and lactate within the lumen 17. Additionally, the colonic inflammation generates extra electron acceptors such as nitrate (NO_3_^-^) and tetrathionate (S_4_O_6_^2-^). These host-derived resources promote the expansion of the facultative anaerobic *Enterobacteriaceae* 20. Our recent study demonstrated that a persistent HFD reduced butyrate concentrations in the colonic contents, leading to impaired mitochondrial activity and metabolic reprogramming of colonocytes 23. This process favors colonic *Proteobacteria* and triggers colonic inflammation. However, it remains to be determined whether EPs regulate the interactions between gut microbiota and colonocytes to mitigate HFD-induced cognitive impairments.

The present study showed that supplementation with EPs effectively ameliorated gut dysbiosis, spine loss, and cognitive behavioral deficits in mice fed with HFD. Based on these results, we further investigated the impact of EPs, which regulate interactions between gut microbiota and colonocytes, on HFD-induced behavioral deficits. Such findings underscore the necessity to further explore the microbial-gut-brain axis and offer new insights into treating diet-induced psychological and neurological disorders.

## Results

### EPs supplementation ameliorated HFD-induced cognitive impairments

**Figure 1:**
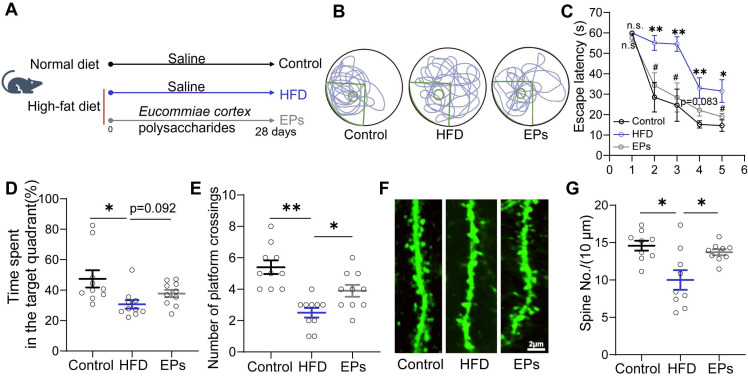
** EPs supplementation improved HFD-induced cognitive deficits.** (A) Schematic illustration of experimental design. Mice were fed with the following diet respectively, Control: normal diet, HFD: a diet containing high fat, EPs: a diet containing high fat, but supplemented with EPs. (B) Representative track plot of spatial memory test (with hidden platform: the green circle). (C) Escape latency to the platform during the training period of the Morris water maze test (n = 10 individuals/group). (D) Percentage of time spent in the target quadrant and (E) number of platform crossings in the probe trial of the Morris water maze test. Representative images of the dendritic spine (F) and summarized data for spine numbers per 10 μm (G) in the DG of the hippocampus in Thy1-YFP mice (n = 9 slices from 3 mice). Scale bars 2 μm in F. Data represent the mean ± SEM. Statistical significance was assessed using a one-way ANOVA followed by Bonferroni's post hoc test. *p < 0.05, **p < 0.01, ***p < 0.001. Notable near-significant differences (0.05 < *p* < 0.1) are indicated in the figures. Notable non-significant (and non-near significant) differences are indicated by “n.s.” in the figures.

To assess the impact of EPs supplementation on spatial cognitive performance in HFD-fed mice, we administered daily EPs supplementation to the mice for a duration of 28 days (Figure 1A). Following this intervention, we conducted the Morris water maze (MWM) tests. During the training phase of the MWM test, we observed that HFD-fed mice exhibited decreased memory retention, as evidenced by a longer escape latency to reach the hidden platform, compared to mice on a normal diet (Figure 1B-C). However, supplementation with EPs to HFD-fed mice resulted in a reduction in escape latency (Figure 1B-C). Additionally, HFD-fed mice displayed deficits in spatial reference memory, as indicated by decreased time spent in the target quadrant and a lower number of platform crossings during the probe test (Figure 1D-E). Nevertheless, these spatial memory impairments were ameliorated by EPs supplementation (Figure 1D-E). We also examined the density of dendritic spines in the dentate gyrus (DG) of the hippocampus in Thy1-YFP mice, as memory formation is closely linked to synapses composed of axon terminals and dendritic spines 24. Analysis of dendritic spine density in the DG revealed that HFD significantly reduced the dendritic spine density (Figure 1F-G). Conversely, mice receiving EPs supplementation exhibited higher dendritic spine density compared to HFD-fed mice, further supporting the cognitive improvement (Figure 1F-G). Consequently, our results highlight the significant ameliorative effect of EPs on HFD-induced spatial cognitive deficits.

### EPs ameliorated HFD-induced gut dysbiosis and colonic impairments

**Figure 2:**
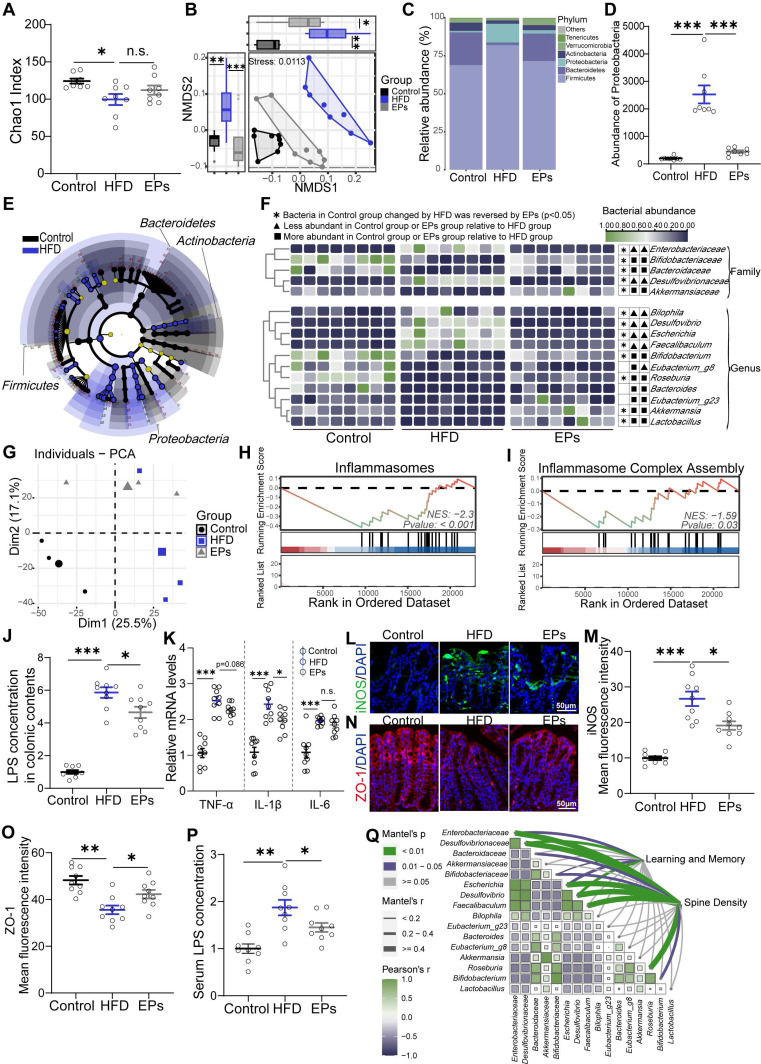
** EPs ameliorated HFD-induced gut dysbiosis and inflammation.** (A) Gut bacterial alpha diversity was estimated by Chao1 index (n = 8 individuals/group). (B) Non-metric Multidimensional Scaling (NMDS) plot showing microbial compositional differences quantified by Bray-Curtis distance. Permutational multivariate analysis of variance (PERMANOVA) by Adonis was used to determine statistical significance. (C) Bar graph of bacterial abundance at the phylum level. (D) Effects of EPs on the abundance of phylum *Proteobacteria* in HFD mice. (E) LEfSe analysis showing bacterial taxa enriched in control and HFD mice, respectively (LDA value = 2.5; *p*‑value < 0.05). (F) Heatmap showing the abundance of bacterial taxa identified by LEfSe. (G) PCA was conducted to assess the colonic transcriptome profiles (n = 3 individuals/group; PERMANOVA by Adonis). (H, I) GSEA showed that EPs inhibited the colonic inflammatory pathway upregulated by HFD. EPs decreased the concentration of LPS in colonic contents (J) and serum (P) of HFD mice (n = 9 for each group). (K) The relative mRNA levels of cytokines (TNF-α, IL-1β and IL-6) in the colonic tissue (n = 9 for each group). (L) Representative immunohistochemical images of iNOS (green) and nuclei labeled with DAPI (blue) in the colon. (N) Representative immunohistochemical images of ZO-1 (red) in the colon. Nuclei were counterstained with DAPI (blue). EPs decreased iNOS protein expression (M) and upregulated the tight junction protein ZO-1 (O) in the colonic tissue of HFD mice (n = 9 slices from 3 mice). (Q) Pairwise comparisons of HFD-altered bacterial taxa are shown, and the color gradient indicates Pearson's correlation coefficient. Relationships between behavioral indicators and dendritic spine density with each bacterial taxon were determined by Mantel's test. Scale bars 50 μm in L and N. In J, K, and P, data were normalized to Control. Data represent the mean ± SEM. Statistical significance was assessed using one-way ANOVA followed by Bonferroni's post hoc test. *p < 0.05, **p < 0.01, ***p < 0.001. Notable non-significant (and non-near significant) differences are indicated by “n.s.” in the figures.

Interventions aimed at modulating the gut microbiota have demonstrated potential in alleviating cognitive impairments in obese individuals, who commonly experience gut dysbiosis 3. The use of dietary polysaccharides as prebiotics to cultivate beneficial colonic bacteria is a prevalent strategy 25. To evaluate the beneficial effects of EPs consumption on the gut microbiota of HFD-fed mice, 16S rRNA gene sequencing was performed. Our findings revealed that persistent HFD reduced bacterial richness (α-diversity) and precipitated a divergent microbial composition compared to mice on a control die (PERMANOVA by Adonis, *p*-value=0.001) (Figure 2A-B). However, supplementation with EPs increased community richness to some extent and reshaped the gut bacterial structure, rendering it more similar to that of the control group rather than the HFD group (EPs vs HFD, PERMANOVA by Adonis, *p*-value=0.009) (Figure 2A-B). Consistent with previous findings 23, HFD substantially elevated the abundance of *Firmicutes* and *Proteobacteria*, while reducing the prevalence of *Bacteroidetes* (Figure 2C-D). Interestingly, EPs administration reversed these changes in the gut microbiota of HFD mice (Figure 2C-D). Further analysis using linear discriminant analysis (LDA) effect size (LEfSe) showed that EPS intervention notably curtailed the bloom of phylum *Proteobacteria* in HFD mice, resulting in a reduction in the abundance of pro-inflammatory genera, such as *Bilophila*, *Desulfovibrio*, and *Escherichia* (Figure 2E-F and Figure S1A). Furthermore, the administration of EPs increased the abundance of SCFA-producing bacteria that were decreased by HFD, including the genus *Akkermansia*, *Bifidobacterium*, and *Roseburia* (Figure 2E-F and Figure S1A).

The increased prevalence of the phylum *Proteobacteria*, a common feature of gut dysbiosis, is known to precipitate colitis and systemic inflammation 26. To investigate the effect of EPs on colonic physiological homeostasis, we conducted a comparison of gene expression profiles of colonic tissue among the Control, HFD, and EPs groups using RNA sequencing. Principal component analysis (PCA) revealed distinct RNA expression patterns across the groups (Figure 2G). The Venn diagram depicted 4932 differently expressed genes (DEGs) between the Control and HFD groups, 2613 DEGs between the HFD and EPs groups, and 1069 DEGs co-regulated by HFD and EPs (Figure S1B). Furthermore, gene set enrichment analysis (GSEA) of DEGs between the HFD and Control groups demonstrated that HFD consumption activated pathways linked to colitis (Figure S1C-E). EPs intervention was observed to influence these processes, such as "Inflammasomes" and "Inflammasome Complex Assembly" (Figure 2H-I). These results were corroborated by the elevated concentration of LPS in the colonic contents (Figure 2J) and heightened mRNA expression of pro-inflammatory cytokines (TNF-α, IL-1β, and IL-6) in the colonic tissue of HFD mice (Figure 2K). Additionally, the immunofluorescent staining results revealed significant insights into colonic tissue characteristics under different treatment conditions. Specifically, the fluorescent intensity of iNOS, a key marker associated with colitis and an inflammatory response, was markedly elevated in the colonic tissue of HFD mice, as illustrated in Figure 2L and M. In conjunction with the altered microbial landscape, especially the excessive growth of phylum *Proteobacteria* which is known to exacerbate colonic inflammation, the treatment with EPs showed a considerable effect in mitigating this inflammatory response (Figure 2H-M). Another crucial aspect examined in this study was the integrity of the intestinal mucosal barrier, which is closely linked to immune homeostasis 27. Tight junction proteins, such as zonula occludens-1 (ZO-1), play a fundamental role in maintaining this barrier. Immunofluorescent staining results showed that EPs supplementation significantly upregulated ZO-1 levels in the colonic tissue of HFD-fed mice (Figure 2N-O). This suggests enhanced tight junction assembly and reinforced barrier integrity, counteracting the increased permeability characteristic of an HFD. The increase in intestinal permeability typically allows bacterial products, such as LPS, to translocate into circulation, triggering systemic inflammation 28. As a result, EPs decreased serum levels of the bacterial-derived LPS in HFD mice (Figure 2P), indicating that EPs improved the integrity of the intestinal barrier and mitigated intestinal permeability. When correlating HFD-altered bacterial taxa with behavioral and physiological indicators, including mice performance in the MWM test and spine density, we found a robust association between the abundance of HFD-altered bacterial taxa and changes in cognitive abilities (Figure 2Q and Figure S1F), implying that gut microbiota are instrumental in mediating the positive cognitive effects of EPs in HFD mice. Collectively, these results indicate that EPs ameliorated the detrimental effects associated with HFD consumption, including gut dysbiosis, barrier dysfunction, and colonic inflammation.

### HFD-induced gut dysbiosis contributed to synaptic engulfment-related cognitive impairments

**Figure 3:**
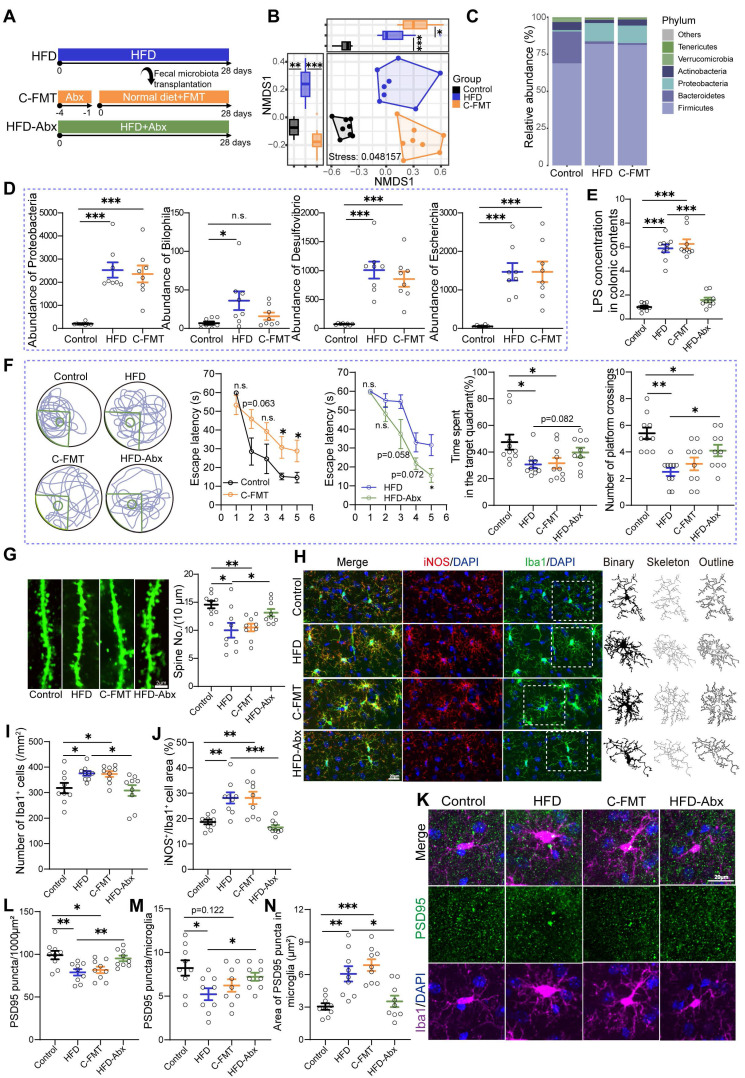
** HFD-induced gut dysbiosis contributed to synaptic engulfment-dependent cognitive impairments.** (A) Schematic diagram of fecal microbiota transplantation (FMT) and antibiotics administration. Feces from HFD mice (with HFD for 28 days) were collected and then used to perform fecal microbiota transplantation into antibiotic-pretreated mice (C-FMT). HFD-fed mice were concurrently treated with an antibiotic cocktail for 28 days (HFD-Abx). (B) Based on the quantification of Bray-Curtis distances, NMDS plots illustrated the similarities in microbial composition between recipient and donor mice (n = 8 individuals/group; PERMANOVA by Adonis). (C) Taxonomic composition at the phylum level. (D) FMT increased the abundance of phylum *Proteobacteria*, genus *Bilophila*, *Desulfovibrio*, and *Escherichia* in normal diet-fed mice. (E) LPS concentration in colonic contents (n = 9 for each group). (F) Representative track plot of spatial memory test (with hidden platform: the green circles) (Left). The escape latency, percentage of time spent in the target quadrant, and number of passes through the platform in the probe trial of the Morris water maze test (Right) (n = 10 individuals/group). (G) Representative images of the dendritic spine (Left) and summarized data for spine numbers per 10 μm in the DG of Thy1-YFP mice (Right) (n = 9 slices from 3 mice). (H) Representative photomicrographs of Iba1 (green) and iNOS (red) expression in the DG (Left). Microglial skeleton and outline (Right). Nuclei were counterstained with DAPI (blue). (I) The microglial density in the DG (n = 9 slices from 3 mice). (J) Percentage of iNOS expressed area to Iba1^+^ microglia expressed area (n = 9 slices from 3 mice). (K) Representative images of Iba1^+^ microglia (magenta) containing PSD95^+^ puncta (green) in the DG. Nuclei were counterstained with DAPI (blue). (L) The density of PSD95^+^ puncta in the DG (n = 9 slices from 3 mice). (M) The number of PSD95^+^ puncta colocalized with Iba1 per microglia in the DG (n = 9 slices from 3 mice). (N) The average area of PSD95^+^ puncta colocalized with Iba1 in the DG (n = 9 slices from 3 mice). Scale bars 2 μm in G, and 20μm in H and K. In E, data were normalized to Control. Data represent the mean ± SEM. Statistical significance was assessed using independent samples t‑test or one-way ANOVA followed by Bonferroni's post hoc test. *p < 0.05, **p < 0.01, ***p < 0.001. Notable near-significant differences (0.05 < *p* < 0.1) are indicated in the figures. Notable non-significant (and non-near significant) differences are indicated by “n.s.” in the figures.

Considering the modulatory effects of EPs on gut microbiota, we wanted to further determine the potential causal relationship between HFD-induced gut dysbiosis and cognitive deficits, which may shed light on how EPs mitigate HFD-induced cognitive decline. FMT was conducted by transferring the fecal microbiota from HFD mice into mice pre-treated with antibiotics (C-FMT) (Figure 3A). The microbial structure of C-FMT mice exhibited greater similarity to that of HFD mice compared to control mice (Figure 3B). Specifically, C-FMT mice showed an increased abundance of *Firmicutes* and *Proteobacteria*, along with a decreased abundance of *Bacteroidetes* (Figure 3C-D and Figure S2A-B). Additionally, the abundance patterns of key bacterial taxa, identified through microbiota-cognition association studies (Figure 2Q and Figure S1F), in C-FMT mice were consistent with those in HFD mice, particularly the expansion of the phylum *Proteobacteria* (Figure 3D). Meanwhile, there was an increase in LPS derived from bacteria (Figure 3E). Thus, these results suggest that the FMT procedure effectively established comparable core microbiomes in C-FMT mice as observed in HFD mice. Subsequent analysis of the behavioral test showed that C-FMT mice exhibited prolonged latency in reaching the platform and reduced time spent in the target quadrant, as well as a decrease in the number of platform crossings (Figure 3F). Furthermore, FMT resulted in a noticeable decrease in dendritic spine density (Figure 3G). To further investigate whether HFD is able to disrupt cognitive function in mice independently of the gut microbiota, we administered HFD-fed mice with a combination of antibiotics to obtain pseudo germ-free mice (H-Abx) (Figure 3A). In comparison to HFD mice, H-Abx mice showed significantly improved spatial memory, as indicated by a decrease in escape latency and an increase in the time spent in the target quadrant and the number of platform crossings (Figure 3F). Accompanying this was a significant increase in the density of neuronal spines (Figure 3G). Systemic inflammation is an essential way for detrimental bacteria to impair cognitive function 29. As expected, mice in the C-FMT group displayed a greater degree of inflammation in the colon by way of increased colonic proinflammatory gene and protein expression compared with those observed in the control mice (Figure S2C-E). Likewise, C-FMT damaged the intestinal barrier by lowering the levels of ZO-1 (Figure S2F-G), which contributed to an increase in the serum concentration of LPS (Figure S2H). H-Abx attenuated the colitis and systemic inflammation induced by HFD (Figure 3E and Figure S2C-E, H), without a significant improvement in barrier damage (Figure S2F-G). These findings strongly support the notion that HFD impairs the cognitive abilities in a gut microbiota-dependent manner and indicate that EPs ameliorate HFD-induced cognitive deficits, which may be attributable to a remodeling effect on the gut microbiota.

**Figure 4:**
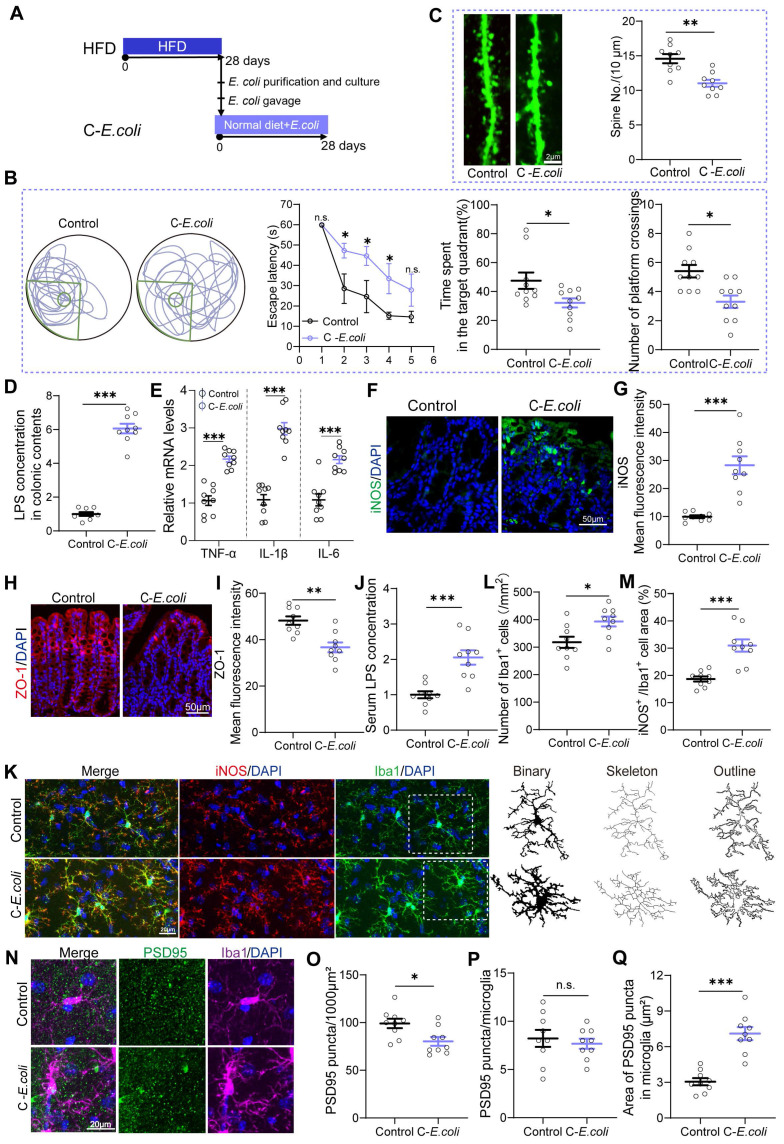
** Overgrowth of *Proteobacteria* as a key factor mediating HFD-induced cognitive impairments in mice.** (A) Indicator strains of* E. coli* isolated from the feces of HFD mice were transplanted into normal diet-fed mice*.* (B) Representative track plot of the Morris water maze test in mice colonized with *E. coli* indicator strains (Left). The escape latency, percentage of time spent in the target quadrant, and the number of passes through the platform in the probe trial of the Morris water maze test (Right) (n = 10 individuals/group). (C) Colonization with *E. coli* resulted in a reduction in the density of dendritic spines in the hippocampus (n = 9 slices from 3 mice). (D) LPS concentration in colonic contents (n = 9 for each group). (E) The mRNA expression of TNF‑α, IL‑1β, and IL‑6 in the colon (n = 9 for each group). (F) Immunofluorescent images of colonic sections stained with iNOS (green) and DAPI (blue). (G) Quantification of iNOS expression levels (n = 9 slices from 3 mice). (H) Immunofluorescent images of colonic sections stained with ZO-1 (red) and DAPI (blue). (I) Quantification of ZO-1 expression levels (n = 9 slices from 3 mice). (J) Serum LPS levels (n = 9 for each group). (K) Representative photomicrographs of Iba1 (green) and iNOS (red) expression in the DG (Left). Microglial skeleton and outline (Right). Nuclei were counterstained with DAPI (blue). (L) The microglial density in the DG (n = 9 slices from 3 mice). (M) Percentage of iNOS expressed area to Iba1 microglia expressed area (n = 9 slices from 3 mice). (N) Representative images of Iba1 microglia (magenta) containing PSD95^+^ puncta (green) and DAPI (blue) in the DG. (O) The density of PSD95^+^ puncta in the DG (n = 9 slices from 3 mice). (P) The number of PSD95^+^ puncta colocalized with Iba1 per microglia in the DG (n = 9 slices from 3 mice). (Q) The average area of PSD95^+^ puncta colocalized with Iba1 in the DG (n = 9 slices from 3 mice). Scale bars 2 μm in C, 50μm in F and H, and 20μm in K and N. In D, E and J, data were normalized to Control. Data represent the mean ± SEM. Statistical significance was compared by independent samples* t*‑test. *p < 0.05, **p < 0.01, ***p < 0.001. Notable non-significant (and non-near significant) differences are indicated by “n.s.” in the figures.

Gut bacteria play a crucial role in modulating brain development and function, partly by affecting the activity of microglia 30. Microglia exhibit changes in density and morphology in response to various forms of neurological insults and can be activated by LPS to hyper-phagocytose neuronal synapses, leading to abnormal neuronal plasticity and cognitive deficits 31. In a resting state, microglia display a ramified morphology characterized by small cell bodies and extensive, finely branched processes. Upon activation, microglia undergo significant morphological changes, including hypertrophy of the cell body and retraction of the processes. In view of the remarkable impact of HFD on the structure of the gut microbiota, we conducted further investigations into the immune response and microglia activity in the hippocampus of HFD mice. Our initial focus centered on several parameters associated with neuroinflammation, including the density and morphology of microglia identified by their characteristic Iba1 immunoreactivity (Iba1^+^ cells) (Figure 3H). To further delineate the type of microglial activation, we performed co-immunostaining using the iNOS antibody, which specifically identifies M1-type, pro-inflammatory microglia (Figure 3H). Consistent with the increased bacteria-derived LPS in serum (Figure 2P), we observed a heightened density of microglia in the DG of the hippocampus in HFD mice compared to those on a standard diet (Figure 4H-I). Additionally, skeleton analysis revealed a "hyper-ramified" activation morphology in microglia from HFD mice, which was demonstrated by an increase in the number of processes, junctions, and total process lengths, as well as a decrease in average process length (Figure S2I-L). To summarize the complexity of the cells, we calculated the fractal dimension. Our findings indicated that microglia in HFD mice exhibited increased complexity and density compared to that in control mice (Figure S2M-N). Furthermore, the levels of iNOS were also elevated in HFD mice with microglial activation, indicating the promotion of a pro-inflammatory phenotype by HFD (Figure 3H and J). To determine the role of changes in gut commensal microbiota in the activation of hippocampal microglia in response to dietary patterns, we analyzed the morphological and immunological characteristics of hippocampal microglia in C-FMT and H-Abx mice. Consistent with the FMT results (Figure 3B-D), C-FMT mice exhibited remarkable hippocampal neuroinflammation, as evidenced by activated microglia and increased iNOS expression (Figure 3H-J). Moreover, HFD-induced neuroinflammation was abolished by antibiotics administration, characterized by suppressed microglia activity and iNOS expression (Figure 3H-J and Figure S2I-N). These results confirmed that gut microbiota is essential for HFD-induced microglial hyperactivation.

Studies have documented postsynaptic density protein-95 positive (PSD95^+^) puncta, which serve as a marker of excitatory synapses, as a synaptic element in microglia-mediated synaptic engulfment 32. We focused on the localization of PSD95^+^ puncta in microglia within the DG. Immunofluorescent staining of PSD95 revealed significant changes associated with diet patterns. Specifically, the density of PSD95^+^ puncta was significantly lower in HFD mice compared to control mice (Figure 3K-L), which was further confirmed by western blot assays (Figure S2O-P). This observation is consistent with the reduction in spine density and cognitive ability in these mice. Furthermore, we assessed the extent of synaptic engulfment by microglia. There was a notable decrease in the density of PSD95^+^ puncta engulfed by microglia in the DG of HFD mice (Figure 3K and M). Interestingly, despite this decrease in density, we observed an increase in the area of PSD95^+^ puncta within microglia (Figure 3K and N). These results suggest that HFD increased the microglial phagocytosis to PSD95 in the hippocampus. A similar pattern of synaptic engulfment and PSD95 expression was observed in recipient mice that received the microbiome from HFD mice (Figure 3K-N and Figure S2O-P). However, clearance of gut microbiota attenuated microglial phagocytosis of synaptic elements and restored PSD95 levels in HFD-fed mice (Figure 3K-N and Figure S2O-P). Taken together, these results highlight the crucial role of gut microbiota in mediating phagocytosis of neuronal synapses by microglia, and suggest that HFD-induced impairments in cognitive may be due, at least in part, to microbiota-triggered neuroinflammation and synaptic phagocytosis.

### *Proteobacteria* expansion as a core factor mediating HFD-induced cognitive deficits

Upon elucidating the fundamental involvement of gut microbiota in HFD-induced cognitive impairments, we aimed to identify the specific bacterial taxa implicated in cognitive deficits within the complexity of gut microbial communities in mice. Correlation analysis revealed a significant association between bacterial taxa from the *Proteobacteria* phylum and cognitive deficits (Figure 2Q and Figure S1F). Therefore, we conducted further investigations into the role of *Proteobacteria* in cognitive deficits by supplementing standard diet-fed mice with *E. coli* indicator strains, isolated from the feces of HFD mice, for a duration of 28 days (Figure 4A). As expected, mice colonized with *E. coli* exhibited impaired cognitive compared to controls, which was evidenced by an increase in escape latency and a significant reduction in the residence time in the target quadrant and the number of platform crossings (Figure 4B). Additionally, colonization with *E. coli* resulted in a reduction in the density of dendritic spines in the DG of the hippocampus (Figure 4C). Furthermore, oral *E. coli* significantly increased the concentration of LPS in the intestinal contents and the expression of pro-inflammatory cytokines and iNOS in the colonic tissue compared to control mice (Figure 4D-G). *E. coli* expansion also disrupted intestinal barrier function in normal-diet mice, as indicated by decreased expression of ZO-1 and increased serum LPS levels (Figure 4H-J). These results highlighted the initiating role of* Proteobacteria* expansion in HFD-induced cognitive deficits.

Moreover, we found that colonization with *E. coli* activated the pro-inflammatory phenotype of microglia in mice, as evidenced by an increase in the density of microglia and the levels of iNOS, as well as morphological abnormalities (Figure 4K-M and Figure S3A-F). Additionally, compared with the Control group, synaptic engulfment was evident in the C-*E. coli* group, as reflected by the increased area of PSD95^+^ puncta in microglia. (Figure 4N and Q). As a consequence, the PSD95 expression was remarkably decreased in DG (Figure 4N-O and Figure S3G-H). Notably, no statistical differences was observed between control and *E. coli*-colonized mice in the density of PSD95^+^ puncta in microglia (Figure 4N and P). The results presented above suggest that colonization of *Proteobacteria* triggered both colonic and systemic inflammation, which in turn contributed to neuroinflammation and synaptic engulfment. These processes played a determinant role in the development of cognitive deficits induced by HFD. Considering the observed inhibitory effect of EPs on the expansion of *Proteobacteria* in HFD-fed mice, we conclude that the decrease of *Proteobacteria* was a necessary precondition for the protective effect of EPs against HFD-induced systemic inflammation, synaptic loss, and cognitive deficits.

### EPs remodeled the metabolism of colonocytes in HFD mice

**Figure 5:**
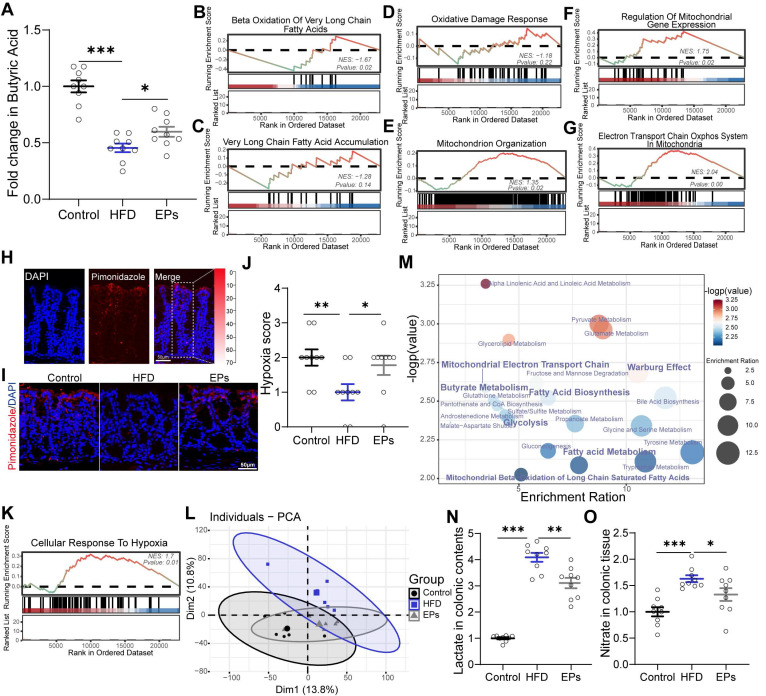
** EPs remodeled the metabolism of colonocytes in HFD mice.** (A) The concentration of butyrate in the colonic contents (n = 9 for each group). (B, C) GSEA showing the enrichment of gene sets for the metabolism of long-chain or very long-chain fatty acids (n = 3 individuals/group). (D) Enriched gene sets of oxidative stress in colonic tissue (n = 3 individuals/group). (E-G) Enriched gene sets of mitochondrial function in colonocytes (n = 3 individuals/group). (H, I) The binding of pimonidazole (red) was used to qualify the oxygen gradient in the colon. Nuclei were counterstained with DAPI (blue). (J) Pimonidazole staining was quantified by scoring blinded sections of the colon (n = 9 slices from 3 mice). (K) EPs restored physiological epithelial hypoxia in the colonic tissue of HFD mice (n = 3 individuals/group). (L) PCA score plots for assessing colonic metabolomic data (n = 6 individuals/group; PERMANOVA by Adonis). (M) The quantitative metabolite set enrichment analysis (qMSEA) method was utilized to determine the colonic metabolic pathways. The concentrations of lactate in colonic contents (N) and nitrate colonic tissue (O) were determined in the colon (n = 9 for each group). Scale bars 50 μm in H and I. In A, N and O, data were normalized to Control, and statistical significance was assessed using a one-way ANOVA followed by Bonferroni's post hoc test. In J, the *p*-value was determined by the Kruskal-Wallis test. Data represent the mean ± SEM. *p < 0.05, **p < 0.01, ***p < 0.001.

The metabolism of colonocytes plays a central role in shaping the gut microbiota 20. Butyrate, derived from obligate anaerobes, activates PPAR-γ and promotes the mitochondrial β-oxidation of SCFAs in colonocytes 33. The metabolism of colonocytes is orientated towards oxidative phosphorylation in the mitochondria, resulting in high epithelial oxygen consumption 34. This helps maintain the anaerobic properties of the colonic lumen to inhibit the growth of facultative anaerobes such as *Proteobacteria*. The expansion of facultative anaerobic* Proteobacteria* is a hallmark of epithelial dysfunction 10. In a recent study, we demonstrated that colonic metabolic reprogramming induced by HFD is responsible for the expansion of *Proteobacteria* 23. Building on this finding, we further explored whether colonic metabolism is involved in the process by which EPs inhibit the overgrowth of *Proteobacteria* induced by HFD. Consistent with the decrease in the abundance of butyrate-producing bacteria, sustained HFD dramatically lowered butyrate concentrations in the colonic contents and PPAR-γ expression in colonic tissue (Figure 5A and Figure S4L-M). However, the administration of EPs effectively reversed these deficits (Figure 5A and Figure S4L-M). Furthermore, GSEA analysis showed that EPs reduced the abnormal increase in long-chain and very long-chain fatty acid metabolism processes in colonocytes of HFD mice (Figure S4A-D and Figure 5B-C). Initiating mitochondrial β-oxidation of long-chain fatty acids leads to higher levels of reactive oxygen species (ROS) generation and impairs mitochondrial function 11, 35, 36. As expected, HFD mice exhibited increased oxidative stress (Figure S4H-I) and impaired mitochondrial activity in colonocytes, as indicated by an increase in "Abnormality of the Mitochondrion" and a decrease in "Mitochondrial RNA Processing" and "Mitochondrial Gene Expression" (Figure S4E-G). This result was further confirmed by the reduction of ATP levels in colonic tissues (Figure S4N). However, EPs ameliorated mitochondrial impairment in colonocytes by impacting the aforementioned processes, including "Oxidative Damage Response", "Mitochondrion Organization" and "Regulation of Mitochondrial Gene Expression" (Figure 5D-F). Additionally, EPs promoted oxidative phosphorylation and ATP production in the mitochondria of HFD mice (Figure 5G and Figure S4N). Based on the restorative effect of EPs on the impaired mitochondrial activity of colonocytes induced by HFD, we conducted additional research on the colonic epithelial oxygenation in HFD mice supplemented with EPs using pimonidazole, an exogenous hypoxia marker (Figure 5H). As shown in Figure 5I and 5J, persistent high-fat intake eliminated epithelial hypoxia, indicating elevated luminal oxygen levels. Conversely, EPs supplementation in HFD mice remodeled physiological hypoxia and mitochondrial function in colonic tissues (Figure 5I-K), representing a potential mechanism by which EPs inhibited the expansion of *Proteobacteria*.

Impaired mitochondrial activity shifted the metabolism of colonocytes towards aerobic glycolysis (Figure S4J), characterized by low oxygen consumption, high lactate release, and increased nitrate synthesis. To examine the remodeling effect of EPs on colonic metabolism in HFD mice, an untargeted metabolomics analysis of colonic tissue was conducted. PCA revealed that the metabolic characteristics of EPs mice bore a greater resemblance to those of control mice in comparison to those of HFD mice (Figure 5L). In accordance with the colonic RNA sequencing results, quantitative metabolite set enrichment analysis (qMSEA) showed that EPs supplementation improved butyrate metabolism and inhibited β-oxidation of long-chain fatty acids in colonocytes, which contributed to rescue HFD-induced mitochondrial dysfunction and the up-regulation of the Warburg effect, a marker of aerobic glycolysis (Figure S4K and Figure 5M). Simultaneously, HFD-induced metabolic reprogramming in colonocytes increased concentrations of lactate in colonic contents and pyruvate and nitrate levels in colonic tissue (Figure 5N-O and Figure S4O). This provided pathogen expansion, particularly the *Proteobacteria*, with host-derived electron acceptors (i.e., nitrate) and carbon sources (i.e., lactate), allowing them to dominate the ecological niche in the gut. In line with the inhibitory effect of *Proteobacteria* expansion, supplementation with EPs reduced lactate levels in colonic contents and pyruvate and nitrate levels in colonic tissue of HFD mice (Figure 5N-O and Figure S4O). These results suggest that EPs remodeled colonocyte metabolism in HFD mice, subsequently decreasing the availability of respiratory substrates for *Proteobacteria*, which may underlie the inhibitory mechanism of EPs against *Proteobacteria* expansion.

### EPs attenuated HFD-induced cognitive deficits by modulating the interaction between gut microbiota and colonocytes

**Figure 6:**
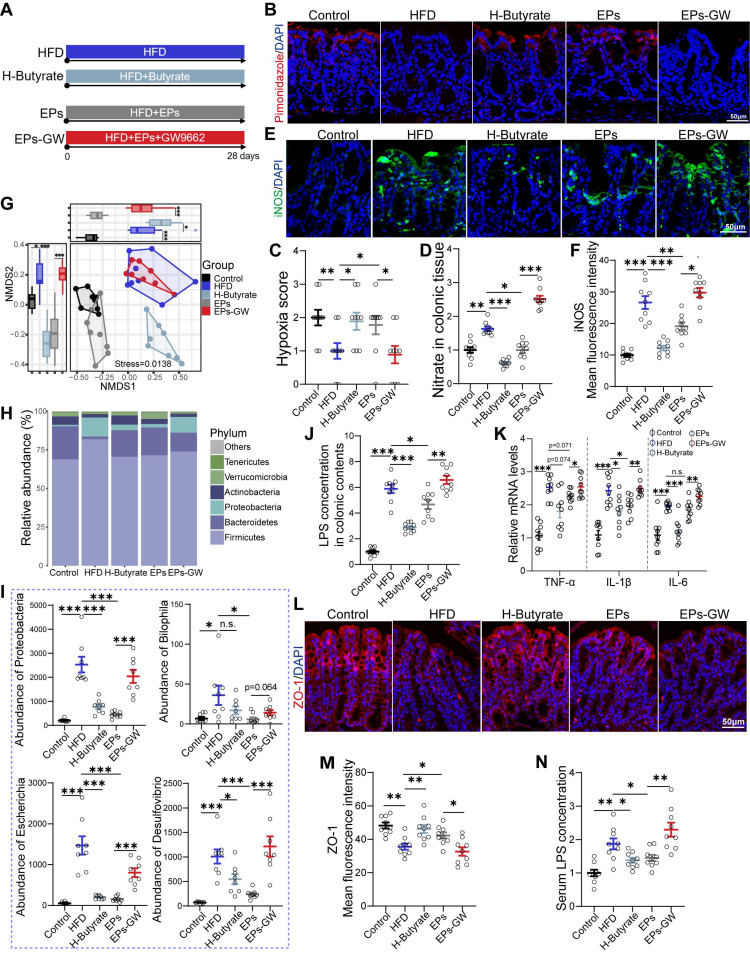
** EPs remodeled gut microbiota-colonocyte interactions to inhibit the expansion of *Proteobacteria* and the following inflammatory response.** (A) To confirm the beneficial effects of increased butyrate production by EPs on cognitive deficits, butyrate was administered to HFD mice in drinking water (H-Butyrate). To further determine whether the amelioration of HFD-induced cognitive deficits by EPs is independent of modulating gut bacteria-colonocyte interactions, we administered EPs mice with the GW9662 (EPs-GW). Immunofluorescent staining for pimonidazole (red) and nuclei (blue) in colonic tissue (B) and quantification of the staining intensities (C) (n = 9 slices from 3 mice). Nitrate concentrations in colonic tissue (D) were determined (n = 9 for each group). (E) Immunofluorescent images of colonic sections stained with iNOS (green) and DAPI (blue). (F) Quantification of iNOS expression levels (n = 9 slices from 3 mice). (G) NMDS based on the Bray-Curtis distance showing the microbiota compositional structure for each group (n = 8 individuals/group; PERMANOVA by Adonis). (H) Gut microbial composition at the phylum level. (I) The abundance of phylum *Proteobacteria*, genus *Bilophila*, *Desulfovibrio*, and *Escherichia*. (J) LPS levels in colonic contents (n = 9 for each group). (K) Colonic TNF-α, IL-1β and IL-6 levels detected by RT-qPCR (n = 9 for each group). (L) Immunofluorescent images of colonic sections stained with ZO-1 (red) and DAPI (blue). (M) Quantification of ZO-1 expression levels (n = 9 slices from 3 mice). (N) Serum LPS levels (n = 9 for each group). Scale bars 50 μm in B. E and L. In D, J, K and N, data were normalized to Control. Data represent the mean ± SEM. In C, the *p*-value was determined by the Kruskal-Wallis test. The rest of the statistical significance was assessed using a one-way ANOVA followed by Bonferroni's post hoc test. *p < 0.05, **p < 0.01, ***p < 0.001. Notable near-significant differences (0.05 < *p* < 0.1) are indicated in the figures. Notable non-significant (and non-near significant) differences are indicated by “n.s.” in the figures.

**Figure 7:**
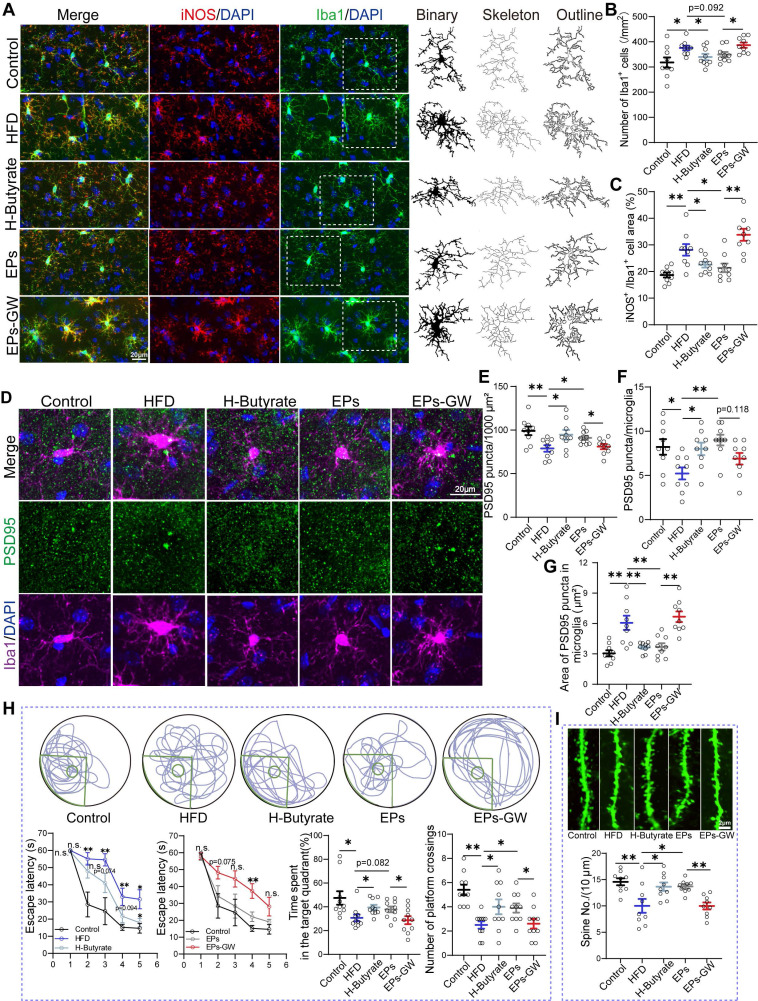
** EPs improved HFD-induced synaptic engulfment-dependent cognitive deficits by remodeling gut microbiota and colonocyte interactions.** (A) Representative photomicrographs of Iba1 (green) and iNOS (red) expression in the DG (Left). Microglial skeleton and outline (Right). Nuclei were counterstained with DAPI (blue). (B) The microglial density in the DG (n = 9 slices from 3 mice). (C) Percentage of iNOS expressed area to Iba1 microglia expressed area (n = 9 slices from 3 mice). (D) Representative images of Iba1 microglia (magenta) containing PSD95^+^ puncta (green) and DAPI (blue) in the DG. (E) The density of PSD95^+^ puncta in the DG (n = 9 slices from 3 mice). (F) The number of PSD95^+^ puncta colocalized with Iba1 per microglia in the DG (n = 9 slices from 3 mice). (G) The average area of PSD95^+^ puncta colocalized with Iba1 in the DG (n = 9 slices from 3 mice). (H) Representative track plots of spatial memory test (with hidden platform: green circles) (Up). The escape latency, percentage of time spent in the target quadrant, and number of passes through the platform in the probe trial of the Morris water maze test (Down) (n = 10 individuals/group). (I) Representative images of dendritic spine (Up) and summarized data for spine numbers per 10 μm (Down) in the DG of Thy1-YFP mice (n = 9 slices from 3 mice). Scale bars 20 μm in A and D, 2μm in I. Data represent the mean ± SEM. Statistical significance was assessed using a one-way ANOVA followed by Bonferroni's post hoc test. *p < 0.05, **p < 0.01, ***p < 0.001. Notable near-significant differences (0.05 < *p* < 0.1) are indicated in the figures. Notable non-significant (and non-near significant) differences are indicated by “n.s.” in the figures.

Given the observation that EPs increased butyrate production in HFD mice, we wanted to investigate the role of this process in mitigating HFD-induced cognitive deficits. For this purpose, HFD mice were provided with butyrate in their drinking water daily for 28 days (H-Butyrate) (Figure 6A). Similar to the metabolic profile of colonocytes observed in EPs-treated mice, butyrate supplementation restored energy metabolism homeostasis and reduced nitrate production in the colonic tissue of HFD mice (Figure 6B-D and Figure S5M-N). Besides serving as a substrate for energy metabolism in colonocytes, butyrate inhibited the expression of iNOS in the colonic tissue (Figure 6E-F). Meanwhile, butyrate supplementation significantly upregulated PPAR-γ expression in colon tissue (Figur S5K-L). Remarkably, butyrate ameliorated HFD-induced gut dysbiosis, as indicated by the distinct gut microbiota structure of H-Butyrate mice compared to HFD mice (PERMANOVA by Adonis, *p*-value=0.006), especially exemplified by the reduced presence of *Proteobacteria* (Figure 6G-I and Figure S5A-B). Meanwhile, butyrate alleviated HFD-induced colitis, as indicated by restoring the integrity of the gut barrier and decreasing LPS levels in the gut lumen and pro-inflammatory cytokines in the colonic tissue (Figure 6J-M). Correspondingly, a decrease in serum LPS levels was observed in H-Butyrate mice (Figure 6N). Furthermore, HFD mice supplemented with butyrate exhibited a notable reduction in both the density and "hyper-ramified" morphology of microglia, along with decreased iNOS expression in the DG of the hippocampus (Figure 7A-C and Figure S5C-H). This observation suggests that butyrate administration ameliorated HFD-induced neuroinflammation in the hippocampus. Simultaneously, butyrate attenuated synapse loss in the DG of HFD mice by inhibiting the excessive engulfment of PSD95 by microglia (Figure 7D-G, I and Figure S5I-J). Importantly, the inhibitory effect of butyrate on synaptic engulfment reversed HFD-induced spatial memory deficits (Figure 7H). These results reveal that modulation of the gut bacteria in mice by EPs, particularly increasing the abundance of butyrate-producing bacteria, was critical in mediating the amelioration of HFD-induced cognitive deficits.

To further determine whether the amelioration of HFD-induced cognitive deficits by EPs is independent of modulating gut bacteria-colonocyte interactions, we administered EPs mice with the GW9662, a PPAR-γ antagonist, to inhibit mitochondrial oxidative phosphorylation in colonocytes (EPs-GW) (Figure 6A). As shown in Figure 6B-D, GW9662 treatment counteracted the ameliorative effect of EPs on colonocyte metabolism, as evidenced by the notable increase in epithelial oxygenation and nitrate production in the colonic tissue of EPs-GW mice in contrast to those in EPs mice. Unsurprisingly, the inhibition of PPARγ signaling by GW9662 resulted in a significant up-regulation of iNOS expression in colonic tissue (Figure 6E-F). 16s profiling showed that the EPs-GW group displayed a distinct shift in bacterial composition relative to the EPs group (PERMANOVA by Adonis, *p*-value = 0.008), characterized by an increase in the abundance of the phylum *Proteobacteria* and the genus *Bilophila*,* Esherichia* and *Desulfovibrio* (Figure 6G-I and Figure S5A-B). Additionally, the beneficial effect of EPs on HFD-induced colitis and barrier damage was abolished by GW9662 treatment, as indicated by increased levels of LPS in the colon content and pro-inflammatory cytokines in the colonic tissue, as well as decreased expression of tight junction protein ZO-1 (Figure 6J-M). In parallel, GW9662 enhanced the concentration of LPS in the serum of EPs mice (Figure 6N).

Gut bacteria transplantation assays highlighted the necessity of HFD-induced expansion of LPS-producing bacteria for hippocampal microglia hyperactivation and dendritic spine loss (Figure 3-4). Consistent with the inhibition of systemic inflammation (Figure 2P), EPs attenuated the density of microglia in the DG of HFD mice to some extent and restored microglial morphology and iNOS expression area to normal levels (Figure 7A-C and Figure S5C-H). We also observed that EPs inhibited HFD-induced PSD95 deposition in microglia and subsequently increased PSD95 levels in the DG (Figure 7D-G and Figure S5I-J). Nevertheless, the beneficial effects of EPs on HFD-induced neuroinflammation and synaptic engulfment were reversed by GW9662 (Figure 7A-G, I and Figure S5C-J). Disturbed the interaction between gut microbiota and colonocytes by GW9662 weakened the protective effect of EPs on HFD-induced spatial memory impairment (Figure 7H). In summary, these results further emphasize the criticality of the interaction between gut microbiota and colonocytes in mediating the improvement of HFD-induced synapse engulfment-dependent cognitive impairments by EPs.

## Discussion

Converging lines of evidence from clinical, epidemiological and animal studies suggest that excessive HFD intake results in widespread deficits in complex behaviors, including cognition 37, 38. Deficits in cognitive function associated with HFD are accompanied by gut dysbiosis and systemic low-grade inflammation, as well as synapse loss in the hippocampus, a brain region related to cognition 3, 37, 39. Consistent with our recent finding 16, results from this study confirmed the presence of cognitive impairments and gut dysbiosis in HFD mice while demonstrating the effectiveness of EPs in mitigating HFD-induced dysfunction.

However, the causal link between the beneficial effects of EPs on cognitive impairments in HFD mice and the remodeling of the gut microbiota or a specific microbial species remains poorly understood. In the present study, we found that persistent HFD led to a decrease in spine density and impaired cognitive functions by disrupting the gut microbiota, particularly by increasing the abundance of *Proteobacteria*. The underlying mechanism may involve the activation of microglia-mediated neuroinflammation and synaptic engulfment, induced by bacteria-derived LPS. Meanwhile, our previous study reported that HFD disrupted mitochondrial bioenergetics, resulting in metabolic reprogramming in colonocytes that facilitated the growth of *Proteobacteria* 23, which is consistent with the current findings. Importantly, this study demonstrates that EPs enhanced the production of butyrate, which modulated the interactions between colonocytes and the gut microbiota, inhibiting the proliferation of *Proteobacteria* and attenuating the inflammatory response along the gut-brain axis.

The hippocampus is widely recognized as one of the brain regions most susceptible to detrimental factors 40. Impaired neuroplasticity is a major cause of behavioral disorders associated with the hippocampus, such as memory loss, anxiety, and depression 41. Multiple studies have shown that behavioral disorders linked to HFD are correlated with compromised synaptic plasticity in the hippocampus 42. Activated microglia, responsible for excessive phagocytosis of synaptic material, contribute to impaired neuroplasticity 31. In this study, we observed a decrease in spine density in the hippocampus of HFD mouse models, potentially due to an increased rate of synaptic pruning (Figure 1F-G). Moreover, we noted an augmented area of PSD95 deposition in Iba1^+^ cells in HFD mice (Figure 3K and N), which suggests either an enhanced phagocytic activity of microglia toward synaptic components or impaired degradation of these proteins by microglia 43-45. Importantly, both scenarios are associated with impaired neural plasticity. Based on this experimental evidence, we hypothesized that hyperactive microglia may play a role in the cognitive deficits observed in HFD mice, specifically concerning neuronal dendritic spines. Remarkably, we found that inhibiting microglia activation with EPs rescued the density of neural dendritic spines and improved cognitive performance in HFD mice (Figure 3H-N and Figure 1B-G). In our study, we primarily utilized the Morris Water Maze Test, a well-established method for evaluating spatial learning and memory. One of the primary advantages of employing multiple behavioral tests is the ability to provide a more comprehensive cognitive profile 46. By incorporating additional tests such as the Y Maze and Novel Object Recognition test, researchers can evaluate different types of memory and cognitive processes. Meanwhile, each behavioral test is associated with distinct neural substrates and mechanisms 47. Using a variety of tests allows researchers to dissect the role of different brain regions and neural circuits in cognitive function. In light of these advantages, we acknowledge the need to incorporate a broader array of behavioral tests in our future studies. This multi-method approach not only enhances the robustness of our findings but also provides a deeper and more nuanced understanding of the cognitive abilities and potential therapeutic effects in our mouse models.

Evidence from studies on gut-brain axis suggests that gut microbiota plays a key role in the link between dietary patterns and cognitive performance 48. Persistent consumption of HFD in mice resulted in the proliferation of pathogenic bacteria, which triggered colonic inflammation to disrupt the intestinal barrier 1, 11. As a result, inflammatory substances like LPS increased in the bloodstream. Peripheral LPS, which is known to induce a depressive mood, has been used experimentally for a long time as a potent agent for the activation of microglia. Intriguingly, aberrant microglia activation causes a deficit in synaptic pruning, leading to spatial cognitive dysfunction 6. Therefore, we proposed that aberrant synaptic phagocytosis by microglia is involved in gut dysbiosis-related cognitive impairment in HFD mice. In the present study, standard diet-fed mice receiving FMT from HFD mice presented LPS-induced systemic low-grade inflammation and excessive microglial phagocytosis of PSD95, as well as cognitive deficits, which were eliminated in HFD mice by antibiotic administration (Figure S2H and Figure 3F-N). These results suggest the crucial role of gut microbiota in mediating phagocytosis of neuronal synapses by microglia and suggest that HFD-induced impairments in cognitive may be due, at least in part, to microbiota-triggered neuroinflammation and synaptic phagocytosis. Recent studies revealed that maternal fiber deprivation has a long-lasting and detrimental effect on offspring microbiome and results in the gut inflammatory response 49. Moreover, a fiber-deficient diet leads to cognitive deficits and synapse loss in the hippocampus through the involvement of gut microbiota and metabolites 50. Previous research by our group showed that EPs supplementation significantly suppressed western diet-induced expansion of *Enterobacteriaceae* and attenuated intestinal and systemic inflammation in mice 16. Similarly, this work suggests that intervention with EPs significantly reduced the abundance of LPS-producing bacteria and the production of LPS, attenuated colonic inflammation and barrier damage, and reduced serum LPS levels in HFD mice (Figure 2). Thus, the ameliorative effect of EPs supplementation on synaptic loss-dependent cognitive impairments may be attributed to the remodeling of the gut microbial profile as well as the restoration of intestinal immune homeostasis.

One common characteristic shared by HFD mice and C-FMT mice is the high prevalence of *Proteobacteria* phylum in the gut (Figure 3D), which was reversed by supplementation with EPs (Figure 2D-F). The expansion of the *Proteobacteria* phylum has been implicated in the development of a variety of metabolic diseases, primarily through the production of LPS, which induces a series of inflammatory events 51. The outgrowth of facultative anaerobic *Enterobacteriaceae* has also been observed in obese individuals exposed to HFD. Previous literature has established a strong association between bacteria-derived LPS and cognitive impairments 52. In the present study, bacterial taxa belonging to the *Proteobacteria* phylum were strongly associated with cognitive deficits in correlation analyses (Figure 2Q and Figure S1F), prompting us to hypothesize that the response of the phylum *Proteobacteria* to dietary components may be pivotal in mediating cognitive impairment induced by HFD. Mice colonized with *E. coli* presented similar cognitive deficits as HFD mice and showed a significant decrease in spine density in the DG (Figure 4B-C). Moreover, we observed that *E. coli* colonization triggered colonic and systemic inflammation in mice, followed by increased permeability of the gut barrier and elevated serum LPS levels (Figure 4D-J). Further analysis revealed that transplantation of *E. coli* enhanced the engulfment of PSD95 by microglia in the DG (Figure 4K-Q), leading to a decrease in the density of dendritic spines (Figure 4C). These results further confirmed the critical role of gut dysbiosis in HFD-induced synaptic engulfment-dependent cognitive impairments. Importantly, the inhibitory effect of EPs on the *Proteobacteria* is extremely probable to be necessary for the protective effect against systemic inflammation, synapse loss, and cognitive deficits in HFD mice.

Intestinal physiological homeostasis is highly dependent on mitochondrial activity, specifically through oxidative phosphorylation, due to the high energy requirements for nutrient transport, rapid epithelial turnover, and maintenance of the epithelial barrier 17. An important advantage of intestinal obligate anaerobes is their ability to digest complex dietary fibers into fermentation products that can be absorbed by the host 53. providing benefits and protecting against intestinal pathogens 33, 54. Among the fermentation metabolites produced by bacteria, butyrate is not only the main energy source for colon cells but also activates the PPAR-γ signaling to promote the metabolism of colonocytes, inducing β-oxidation of fatty acids in mitochondria, which is crucial for maintaining physiological hypoxia in the colon 33. With this mechanism, the colonic epithelium ensures the predominance of beneficial anaerobic microorganisms, thus maintaining intestinal homeostasis 10. Therefore, metabolic homeostasis of colonocytes plays a pivotal role in forming beneficial microbiota and facilitating microbiota-nourishing immunity 34. However, dietary changes, especially the intake of diets rich in saturated long-chain fatty acids, have been shown to significantly impair colonic mitochondrial function 17. Increased intake of high-saturated fatty acids may directly affect the oxidative capacity of colonocytes, damaging mitochondrial activity by triggering the generation of hydrogen peroxide in mitochondria 36, 55.

Transcriptomic analysis reveals that HFD increased the β-oxidation of long-chain and very long-chain fatty acids in the colonocytes (Figure S4A-D). This process leads to ROS generation and impairments of mitochondrial function 11, 55, 56. Similarly, we observed that persistent HFD damaged mitochondrial function (Figure S4E-G), resulting in increased colonic epithelial oxygenation and enhanced oxygen availability in the colonic lumen (Figure 5H-J). The switch to long-chain and very long-chain fatty acids as substrates for energy metabolism in colonocytes may be attributed to reduced production of bacteria-derived SCFAs. Butyrate-activated PPAR-γ signaling represses the transcription of pro-inflammatory genes, including the iNOS gene 57. in addition to regulating mitochondrial functions. Consistent with decreased butyrate production in the intestinal lumen, iNOS synthesis was increased in the colonic tissues of HFD mice, leading to increased nitrate accessibility in the colonic lumen (Figure 6D-F). The impairments of mitochondrial activity and upregulation of inflammatory signals cause a shift from oxidative phosphorylation to anaerobic glycolysis in colonocytes, characterized by low oxygen consumption, high nitrate generation, and high lactate release. These host-derived resources have been the driving force behind the expansion of facultative anaerobic pathogens 20, particularly *Proteobacteria* 10. Continuous oral antibiotic administration disrupts the intestinal endo-environmental homeostasis associated with the microbiota 58, 59, while supplementing antibiotic-treated mice with high plant fiber or directly activating signaling downstream of butyrate helps restore physiological hypoxia at the surface of the intestinal mucosa 9, 54, 60. Consistent with previous studies, supplementation with EPs or butyrate in HFD mice significantly restored the hypoxia in the colonic epithelium and diminished nitrate generation (Figure 6B-D), thereby decreasing the abundance of *Proteobacteria* (Figure 6I), which contributed to the suppression of the systemic low-grade inflammatory response (Figure 6N). Therefore, the increase in phylum *Proteobacteria* observed in HFD mice is a consequence of the long- or very long-chain fatty acid β-oxidation-induced deterioration of the intestinal epithelial ability to maintain anaerobically-driven intestinal homeostasis. Notably, improvement in cognitive that resulted from EPs supplementation in HFD mice was eliminated by the PPAR-γ antagonist GW9662, further confirming that EPs ameliorate HFD-associated dysfunction primarily by modulating the interaction of gut microbiota with colonocytes.

Our study underscores the complexity of gut-brain related cognitive impairment, emphasizing the multifactorial nature of its pathogenesis. We acknowledge that while the *Proteobacteria*-induced inflammation pathway is a significant factor, it is not the only mechanism at play. Recent research has highlighted the critical role of the complement system, specifically the C3/CR3 signaling pathway, in the pathogenesis of cognitive impairment 61, 62. Complement C3, a central component of the complement cascade, can bind to the complement receptor 3 (CR3) on microglia, the resident immune cells of the brain. This interaction is pivotal in the process of synaptic pruning, where microglia eliminate excess or weak synaptic connections during development and in response to neural damage. Aberrant activation of the C3/CR3 signaling pathway has been linked to excessive synaptic pruning, leading to synaptic loss and cognitive deficits 61. Studies have demonstrated that increased levels of complement C3 in the brain correlate with elevated microglial activity and subsequent synaptic degradation, particularly in conditions of neuroinflammation 61, 63. This pathway becomes especially relevant in the context of gut-brain interactions, where gut-derived inflammatory signals can upregulate complement components, exacerbating neural damage and cognitive decline 64. To provide a more comprehensive understanding, future research should incorporate detailed analyses of the complement system components and microglial activation states in response to gut microbiota modulation. These investigations will help delineate the precise molecular pathways through which gut-derived signals influence brain health and cognitive outcomes.

## Conclusions

Overall, these findings suggest that EPs could effectively ameliorate neuroinflammation and cognitive deficits in HFD-fed mice by modulating colonocyte-gut microbiota interactions. Mechanistic studies revealed that HFD triggered excessive microglial phagocytosis of DG synapses in a gut microbiota-dependent manner, which in turn caused spine loss-related cognitive deficits in mice. However, EPs alleviated mitochondrial dysfunction-mediated metabolic disturbances in colonocytes of mice with HFD by increasing the abundance of butyrate-producing bacteria. These effects favored the inhibition of *Proteobacteria* proliferation in the colon and attenuated the deleterious effects of HFD. The present study contributes to our understanding of the mechanisms underlying cognitive dysfunction associated with HFD, particularly the impact of host commensal microorganisms on microglia function in the CNS. Moreover, it provides the first evidence that EPs can ameliorate diet-induced dysfunction by modulating the interactions between individual gut microbiota and colonocytes, thus offering a theoretical basis for the development of preventive therapeutic strategies in which polysaccharides manipulate the gut microbiota for the treatment of neurological disorders.

## Material and methods

### Purification and characterization of EPs

The methods for extracting and characterizing polysaccharides were described in our previous work 65.

### Mice

Male C57BL/6 mice (4 weeks old) were purchased from SIPEIFU Biotechnology Co., Ltd. Thy1-YFP mouse lines were kind gifts from Prof Zhang, School of Life Sciences, Lanzhou University. Mice were kept under pathogen-free conditions with adequate food and water in the animal laboratory. All experiments were conducted at the College of Veterinary Medicine, Northwest A&F University and were performed following the recommended guidelines from the Administration of Affairs Concerning Experimental Animals (Ministry of Science and Technology, China).

### Experimental setup

#### Modeling and EPs administration

Mice were randomized into the standard diet group (Control; #XTHF0045-1-C), the high-fat diet group (HFD; #XTHF0045-1), and the high-fat diet supplemented with EPs group (EPs; high-fat diet plus daily gavage of EPs 400 mg/kg). Standard feed (10% of calories from fat, 17% of calories from protein, and 73% of calories from carbohydrate) and high-fat feed (63% of calories from fat, 17% of calories from protein, and 20% of calories from carbohydrate) were purchased from Jiangsu Xietong Medicine Bioengineering Co Ltd (Jiangsu, China). The duration of the dietary intervention in the three groups of mice was 28 days.

#### FMT experiment

To investigate the causal role of gut microbiota in HFD-induced cognitive impairments, we performed FMT. The protocol was consistent with our previous report 23. Prior to FMT, in order to massively deplete the microbiota, an antibiotic cocktail (Abx) was administered to the drinking water of standard-diet mice for 3 consecutive days at the following concentrations: metronidazole (100 mg/L), penicillin (100 mg/L), neomycin (100 mg/L), vancomycin (50 mg/L), and streptomycin (50 mg/L) 66. For the preparation of FMT materials, fresh feces were collected from mice on HFD for 28 days and immediately pooled, mixed and homogenized with sterile PBS (1 g feces/10 mL PBS). The mixture was centrifuged at 500 rpm for 5 min at 4°C, and the supernatant was collected. Subsequently, antibiotic-pretreated mice were given the supernatants (150 µl) orally alone once a day for 28 days (C-FMT). In addition, the mice received HFD while being treated with antibiotics to eliminate gut bacteria for 28 days (H-Abx). All antibiotics were purchased from Aladdin, China.

#### *E. coli* colonization experiment

To investigate the role of the *Proteobacteria* phylum expansion in HFD-induced cognitive deficits, *E. coli* was isolated from the feces of HFD mice as an indicator strain for subsequent transplantation experiments. Methods for isolation, purification, and enrichment of *E. coli* were described in previous reports 23. Briefly, fresh feces of HFD mice (0.2 g) were collected, dissolved in sterile PBS, and then isolated and cultured on *E. coli* chromogenic medium (HB7001; Hopebio Co., Qingdao, China). Identification of the isolated colonies was performed using 16S rRNA sequencing and API assays to select *E. coli* indicator strains. Indicated strains were enriched using Luria-Bhertani (LB) broth at 36 °C with shaking. Bacterial cells were collected by centrifugation (5000 g, 4°C, 20 min), then washed and resuspended in saline. The final concentration of *E. coli* was approximated as 1 × 10^8^ CFU/mL in the saline resuspension. The standard diet-fed mice were then given 150 µl of E. coli suspension by gavage daily for 28 consecutive days (C-*E. coli*).

#### Butyrate and GW9662 treatment experiment

For treatment with butyrate, sodium butyrate (Aladdin, China) was added to the drinking water of HFD mice at 0.1 M for 28 days (H-Butyrate). For the treatment with PPAR-γ antagonist GW9662, EPs mice were treated with 5 mg/kg/day of GW9662 (Aladdin, China) intraperitoneally (EPs-GW).

### Morris water maze

At the end of the intervention, some mice were tested in the Morris water maze (MWM) test, which is used to assess spatial cognitive. Experimental methods were performed based on an established protocol 67.

### Immunofluorescent staining, image acquisition and analysis

Mice were perfused with 0.9% saline followed by 4% paraformaldehyde (PFA). The fixed brain was sliced into 50 μm sections with the vibratome (VT1000S, Leica Microsystems, Wetzlar, Germany). Colonic tissue was embedded in paraffin and sliced into 5-μm-thick slides. For hypoxia staining, mice were orally administered 60 mg/kg pimonidazole HCl (Hypoxyprobe^TM^-1 kit, Hypoxyprobe) 1h before sacrifice. For immunohistochemistry, sections were incubated overnight at 4°C with primary antibodies diluted in blocking solution (1% normal goat serum, 0.3% Triton-X, 2% bovine serum albumin in phosphate buffer). Primary antibodies were as follows: rabbit anti-Iba1 (1:1000, Abcam), mouse anti-PSD95 (1:1000, Abcam), mouse anti-iNOS (1:1000, Brand), mouse anti-pimonidazole monoclonal IgG1 (Hypoxyprobe^TM^-1 kit, Hypoxyprobe) and anti-ZO-1 (1:500, Thermo Fisher Scientific). After that, the primary antibody was removed by washing the sections 3 times in PB. The sections were then incubated in species-specific secondary antibodies (1:500 for all) for 3 h at room temperature. 4′,6-diamidino-2-phenylindole (DAPI) (10 μg/ml, Abcam) was used to counterstain nuclei. After washing, sections were mounted on glass slides with Fluorescence Mounting Medium (Dako, Denmark) and covered with micro coverslips. The images were acquisited with a laser scanning confocal microscope (FV3000, Olympus, Japan). Quantification of positive cells was performed using ImageJ analysis software (version 1.52). To analyze microglial density and morphology, microglia were imaged using FV3000 with a 20x objective, recording layers every 0.2 μm. Using a method adapted from Young et al.^68^, microglial morphology was measured in all cells.

### Colonic tissue RNA extraction and RT-qPCR

Mice were anesthetized with 0.56 % (v/v) pentobarbital sodium (Sigma, 10 mg/ml) and sacrificed by eyeball extirpating, and biological samples were collected for further analysis. RNA extraction and reverse transcription-quantitative polymerase chain reaction (RT-qPCR) were conducted in accordance with a previous report 65. More details are provided in the Supporting Information. Primers used in RT-qPCR are listed in Table S1.

### 16S rRNA microbiome sequencing

Colonic contents were collected and rapidly frozen in liquid nitrogen. Samples were processed by Magigene Technology Co., Ltd (Guangzhou, China) for DNA extraction, amplification and sequencing. Primer sequences and data processing steps are provided in the Supporting Information.

### Colonic tissue transcriptome profiling

Colonic tissue was collected and rapidly frozen in liquid nitrogen. Samples were processed by Seqhealth Technology Co., Ltd. (Wuhan, China) for RNA-seq processing. Methods for colon tissue genomic DNA extraction, sequencing, and bioinformatics analysis are provided in the Supporting Information.

### Colonic tissue metabolomics

The procedures of sample extraction and instrumental analysis were performed according to the previous protocol 23. More details are provided in the Supporting Information.

### Butyrate and lactate measurements

Determination of butyrate and lactate in colonic contents as previously reported 69, 70. More details are provided in the Supporting Information.

### Nitrate measurements

Nitrate levels in colonic tissues were measured using the Total Nitrate Assay Kit (S0023, Beo Tianmei Biotechnology Co., Ltd., China) following the manufacturer's instructions.

### Quantification of E. coli abundance

The abundance of *E. coli* was determined using the Escherichia Coli Probe PCR Kit (Xin-Yu Biotechnology Co., Ltd, Shanghai, China) according to the manufacturer's instructions.

### Statistical analysis

Statistical procedures and graphs were conducted with SPSS (v.25.0) and GraphPad Prism (v.9.0), respectively. For two group comparisons, Student's *t*-test was applied. One-way analysis of variance (ANOVA) followed by Bonferroni's post hoc test was used for multiple group comparisons and correction. The statistical significance of changes in hypoxia levels was determined using a non-parametric test (Wilcoxon test). Data are shown as individual values or expressed as the mean ± standard error of mean (SEM), and significance levels are indicated as *p < 0.05, **p < 0.01, ***p < 0.001 and not significant (n.s.). *P values* are not provided as exact values when they less than 0.0001. Notable near-significant differences (0.05 < *p* < 0.1) are indicated in the figures.

## Supplementary Material

Supplementary experimental section, figures and table.

## Abbreviations

HFD: High-fat diet
EPs: *Eucommiae cortex* polysaccharides
FMT: Fecal microbiota transplantation
*E. coli*: *Escherichia coli*
CNS: Central nervous system
LPS: Lipopolysaccharide
SCFAs: Short-chain fatty acids
PPAR-γ: Peroxisome proliferator-activated receptor-γ
MWM: Morris water maze
DG: Dentate gyrus
LEfSe: Linear discriminant analysis effect size
PCA: Principal component analysis
DEGs: Differently expressed genes
GSEA: Gene set enrichment analysis
ZO-1: Zonula occludens-1
PSD95: Postsynaptic density protein-95 positive
ROS: Reactive oxygen species
qMSEA: quantitative metabolite set enrichment analysis

## References

[1] Kang SS, Jeraldo PR, Kurti A, Miller MEB, Cook MD, Whitlock K (2014). Diet and exercise orthogonally alter the gut microbiome and reveal independent associations with anxiety and cognition. Mol Neurodegener.

[2] Wang L, Gong Z, Zhang X, Zhu F, Liu Y, Jin C (2020). Gut microbial bile acid metabolite skews macrophage polarization and contributes to high-fat diet-induced colonic inflammation. Gut microbes.

[3] Bruce-Keller AJ, Salbaum JM, Luo M, Blanchard E, Taylor CM, Welsh DA (2015). Obese-type gut microbiota induce neurobehavioral changes in the absence of obesity. Biol Psychiatry.

[4] Hickman S, Izzy S, Sen P, Morsett L, El Khoury J (2018). Microglia in neurodegeneration. Nat Neurosci.

[5] Zhang M, Chen H, Zhang W, Liu Y, Ding L, Gong J (2023). Biomimetic Remodeling of Microglial Riboflavin Metabolism Ameliorates Cognitive Impairment by Modulating Neuroinflammation. Adv Sci (Weinh).

[6] Ballasch I, García-García E, Vila C, Pérez-González A, Sancho-Balsells A, Fernández J (2023). Ikzf1 as a novel regulator of microglial homeostasis in inflammation and neurodegeneration. Brain Behav Immun.

[7] Margolis KG, Cryan JF, Mayer EA (2021). The Microbiota-Gut-Brain Axis: From Motility to Mood. Gastroenterology.

[8] Yoo W, Zieba JK, Foegeding NJ, Torres TP, Shelton CD, Shealy NG (2021). High-fat diet-induced colonocyte dysfunction escalates microbiota-derived trimethylamine N-oxide. Science.

[9] Lee J-Y, Cevallos SA, Byndloss MX, Tiffany CR, Olsan EE, Butler BP (2020). High-Fat Diet and Antibiotics Cooperatively Impair Mitochondrial Bioenergetics to Trigger Dysbiosis that Exacerbates Pre-inflammatory Bowel Disease. Cell Host Microbe.

[10] Litvak Y, Byndloss MX, Tsolis RM, Bäumler AJ (2017). Dysbiotic Proteobacteria expansion: a microbial signature of epithelial dysfunction. Curr Opin Microbiol.

[11] Zeng N, Wu F, Lu J, Li X, Lin S, Zhou L (2024). High-fat diet impairs gut barrier through intestinal microbiota-derived reactive oxygen species. Sci China Life Sci.

[12] Martinez-Medina M, Denizot J, Dreux N, Robin F, Billard E, Bonnet R (2014). Western diet induces dysbiosis with increased E coli in CEABAC10 mice, alters host barrier function favouring AIEC colonisation. Gut.

[13] Liu H, Liao C, Wu L, Tang J, Chen J, Lei C (2022). Ecological dynamics of the gut microbiome in response to dietary fiber. ISME J.

[14] Holscher HD (2017). Dietary fiber and prebiotics and the gastrointestinal microbiota. Gut microbes.

[15] Liu A, Zhang M, Wu Y, Zhang C, Zhang Q, Su X (2023). ASPS Exhibits Anti-Rheumatic Effects by Reprogramming Gut Microbiota and Increasing Serum γ-Glutamylcysteine Level. Adv Sci (Weinh).

[16] Sun P, Wang M, Li Z, Wei J, Liu F, Zheng W (2022). Eucommiae cortex polysaccharides mitigate obesogenic diet-induced cognitive and social dysfunction via modulation of gut microbiota and tryptophan metabolism. Theranostics.

[17] Guerbette T, Boudry G, Lan A (2022). Mitochondrial function in intestinal epithelium homeostasis and modulation in diet-induced obesity. Mol Metab.

[18] Donohoe DR, Garge N, Zhang X, Sun W, O'Connell TM, Bunger MK (2011). The microbiome and butyrate regulate energy metabolism and autophagy in the mammalian colon. Cell Metab.

[19] Topping DL, Clifton PM (2001). Short-chain fatty acids and human colonic function: roles of resistant starch and nonstarch polysaccharides. Physiol Rev.

[20] Litvak Y, Byndloss MX, Bäumler AJ (2018). Colonocyte metabolism shapes the gut microbiota. Science.

[21] Roediger WE (1980). Role of anaerobic bacteria in the metabolic welfare of the colonic mucosa in man. Gut.

[22] Ndeh D, Gilbert HJ (2018). Biochemistry of complex glycan depolymerisation by the human gut microbiota. FEMS Microbiol Rev.

[23] Sun P, Wang M, Liu Y-X, Li L, Chai X, Zheng W (2023). High-fat diet-disturbed gut microbiota-colonocyte interactions contribute to dysregulating peripheral tryptophan-kynurenine metabolism. Microbiome.

[24] Nguyen PT, Dorman LC, Pan S, Vainchtein ID, Han RT, Nakao-Inoue H (2020). Microglial Remodeling of the Extracellular Matrix Promotes Synapse Plasticity. Cell.

[25] Koropatkin NM, Cameron EA, Martens EC (2012). How glycan metabolism shapes the human gut microbiota. Nat Rev Microbiol.

[26] Shin N-R, Whon TW, Bae J-W (2015). Proteobacteria: microbial signature of dysbiosis in gut microbiota. Trends Biotechnol.

[27] Peterson LW, Artis D (2014). Intestinal epithelial cells: regulators of barrier function and immune homeostasis. Nat Rev Immunol.

[28] Zhao C, Bao L, Qiu M, Wu K, Zhao Y, Feng L (2022). Commensal cow Roseburia reduces gut-dysbiosis-induced mastitis through inhibiting bacterial translocation by producing butyrate in mice. Cell Rep.

[29] Leigh S-J, Morris MJ (2020). Diet, inflammation and the gut microbiome: Mechanisms for obesity-associated cognitive impairment. Biochim Biophys Acta Mol Basis Dis.

[30] Abdel-Haq R, Schlachetzki JCM, Glass CK, Mazmanian SK (2019). Microbiome-microglia connections via the gut-brain axis. J Exp Med.

[31] Cao P, Chen C, Liu A, Shan Q, Zhu X, Jia C (2021). Early-life inflammation promotes depressive symptoms in adolescence via microglial engulfment of dendritic spines. Neuron.

[32] Hong S, Beja-Glasser VF, Nfonoyim BM, Frouin A, Li S, Ramakrishnan S (2016). Complement and microglia mediate early synapse loss in Alzheimer mouse models. Science.

[33] Byndloss MX, Olsan EE, Rivera-Chávez F, Tiffany CR, Cevallos SA, Lokken KL (2017). Microbiota-activated PPAR-γ signaling inhibits dysbiotic Enterobacteriaceae expansion. Science.

[34] Shelton CD, Byndloss MX (2020). Gut Epithelial Metabolism as a Key Driver of Intestinal Dysbiosis Associated with Noncommunicable Diseases. Infect Immun.

[35] Chen D, Li X, Zhang L, Zhu M, Gao L (2018). A high-fat diet impairs mitochondrial biogenesis, mitochondrial dynamics, and the respiratory chain complex in rat myocardial tissues. J Cell Biochem.

[36] Cardoso AR, Kakimoto PAHB, Kowaltowski AJ (2013). Diet-sensitive sources of reactive oxygen species in liver mitochondria: role of very long chain acyl-CoA dehydrogenases. PLoS One.

[37] Zhuang H, Yao X, Li H, Li Q, Yang C, Wang C (2022). Long-term high-fat diet consumption by mice throughout adulthood induces neurobehavioral alterations and hippocampal neuronal remodeling accompanied by augmented microglial lipid accumulation. Brain Behav Immun.

[38] Vagena E, Ryu JK, Baeza-Raja B, Walsh NM, Syme C, Day JP (2019). A high-fat diet promotes depression-like behavior in mice by suppressing hypothalamic PKA signaling. Transl Psychiatry.

[39] Henn RE, Elzinga SE, Glass E, Parent R, Guo K, Allouch AA (2022). Obesity-induced neuroinflammation and cognitive impairment in young adult versus middle-aged mice. Immun Ageing.

[40] Tsai S-F, Hsu P-L, Chen Y-W, Hossain MS, Chen P-C, Tzeng S-F (2022). High-fat diet induces depression-like phenotype via astrocyte-mediated hyperactivation of ventral hippocampal glutamatergic afferents to the nucleus accumbens. Mol Psychiatry.

[41] Neves G, Cooke SF, Bliss TVP (2008). Synaptic plasticity, memory and the hippocampus: a neural network approach to causality. Nat Rev Neurosci.

[42] Hao S, Dey A, Yu X, Stranahan AM (2016). Dietary obesity reversibly induces synaptic stripping by microglia and impairs hippocampal plasticity. Brain Behav Immun.

[43] Appel JR, Ye S, Tang F, Sun D, Zhang H, Mei L (2018). Increased Microglial Activity, Impaired Adult Hippocampal Neurogenesis, and Depressive-like Behavior in Microglial VPS35-Depleted Mice. J Neurosci.

[44] Benetatos J, Bennett RE, Evans HT, Ellis SA, Hyman BT, Bodea L-G (2020). PTEN activation contributes to neuronal and synaptic engulfment by microglia in tauopathy. Acta Neuropathol.

[45] Kim HJ, Cho MH, Shim WH, Kim JK, Jeon EY, Kim DH (2017). Deficient autophagy in microglia impairs synaptic pruning and causes social behavioral defects. Mol Psychiatry.

[46] Turner BM, Rodriguez CA, Norcia TM, McClure SM, Steyvers M (2016). Why more is better: Simultaneous modeling of EEG, fMRI, and behavioral data. NeuroImage.

[47] Robie AA, Hirokawa J, Edwards AW, Umayam LA, Lee A, Phillips ML (2017). Mapping the Neural Substrates of Behavior. Cell.

[48] Wang D, Wang L, Han L, Wang B, Shi R, Ye J (2023). Leucine-Restricted Diet Ameliorates Obesity-Linked Cognitive Deficits: Involvement of the Microbiota-Gut-Brain Axis. J Agric Food Chem.

[49] Zou J, Ngo VL, Wang Y, Wang Y, Gewirtz AT (2023). Maternal fiber deprivation alters microbiota in offspring, resulting in low-grade inflammation and predisposition to obesity. Cell Host Microbe.

[50] Shi H, Ge X, Ma X, Zheng M, Cui X, Pan W (2021). A fiber-deprived diet causes cognitive impairment and hippocampal microglia-mediated synaptic loss through the gut microbiota and metabolites. Microbiome.

[51] Shi Y, Zou Y, Xiong Y, Zhang S, Song M, An X (2021). Host Gasdermin D restrains systemic endotoxemia by capturing Proteobacteria in the colon of high-fat diet-feeding mice. Gut microbes.

[52] Du L, Lei X, Wang J, Wang L, Zhong Q, Fang X (2022). Lipopolysaccharides derived from gram-negative bacterial pool of human gut microbiota promote inflammation and obesity development. Int Rev Immunol.

[53] den Besten G, van Eunen K, Groen AK, Venema K, Reijngoud D-J, Bakker BM (2013). The role of short-chain fatty acids in the interplay between diet, gut microbiota, and host energy metabolism. J Lipid Res.

[54] Rivera-Chávez F, Zhang LF, Faber F, Lopez CA, Byndloss MX, Olsan EE (2016). Depletion of Butyrate-Producing Clostridia from the Gut Microbiota Drives an Aerobic Luminal Expansion of Salmonella. Cell Host Microbe.

[55] Kakimoto PAHB, Tamaki FK, Cardoso AR, Marana SR, Kowaltowski AJ (2015). H2O2 release from the very long chain acyl-CoA dehydrogenase. Redox Biol.

[56] Egnatchik RA, Leamy AK, Noguchi Y, Shiota M, Young JD (2014). Palmitate-induced activation of mitochondrial metabolism promotes oxidative stress and apoptosis in H4IIEC3 rat hepatocytes. Metabolism.

[57] Li M, Pascual G, Glass CK (2000). Peroxisome proliferator-activated receptor gamma-dependent repression of the inducible nitric oxide synthase gene. Mol Cell Biol.

[58] Kelly CJ, Zheng L, Campbell EL, Saeedi B, Scholz CC, Bayless AJ (2015). Crosstalk between Microbiota-Derived Short-Chain Fatty Acids and Intestinal Epithelial HIF Augments Tissue Barrier Function. Cell Host Microbe.

[59] Zarrinpar A, Chaix A, Xu ZZ, Chang MW, Marotz CA, Saghatelian A (2018). Antibiotic-induced microbiome depletion alters metabolic homeostasis by affecting gut signaling and colonic metabolism. Nat Commun.

[60] Cevallos SA, Lee J-Y, Velazquez EM, Foegeding NJ, Shelton CD, Tiffany CR (2021). 5-Aminosalicylic Acid Ameliorates Colitis and Checks Dysbiotic Escherichia coli Expansion by Activating PPAR-γ Signaling in the Intestinal Epithelium. mBio.

[61] Li C, Liu B, Xu J, Jing B, Guo L, Wang L (2023). Phloretin decreases microglia-mediated synaptic engulfment to prevent chronic mild stress-induced depression-like behaviors in the mPFC. Theranostics.

[62] Hong S, Beja-Glasser VF, Nfonoyim BM, Frouin A, Li S, Ramakrishnan S (2016). Complement and microglia mediate early synapse loss in Alzheimer mouse models. Science (New York, NY).

[63] Maier M, Peng Y, Jiang L, Seabrook TJ, Carroll MC, Lemere CA (2008). Complement C3 deficiency leads to accelerated amyloid beta plaque deposition and neurodegeneration and modulation of the microglia/macrophage phenotype in amyloid precursor protein transgenic mice. The Journal of neuroscience: the official journal of the Society for Neuroscience.

[64] Hao W, Ma Q, Wang L, Yuan N, Gan H, He L (2024). Gut dysbiosis induces the development of depression-like behavior through abnormal synapse pruning in microglia-mediated by complement C3. Microbiome.

[65] Wang M, Sun P, Li Z, Li J, Lv X, Chen S (2023). Eucommiae cortex polysaccharides attenuate gut microbiota dysbiosis and neuroinflammation in mice exposed to chronic unpredictable mild stress: Beneficial in ameliorating depressive-like behaviors. J Affect Disord.

[66] Li D, Feng Y, Tian M, Ji J, Hu X, Chen F (2021). Gut microbiota-derived inosine from dietary barley leaf supplementation attenuates colitis through PPARγ signaling activation. Microbiome.

[67] Wang X, Wang Z, Cao J, Dong Y, Chen Y (2023). Gut microbiota-derived metabolites mediate the neuroprotective effect of melatonin in cognitive impairment induced by sleep deprivation. Microbiome.

[68] Young K, Morrison H Quantifying Microglia Morphology from Photomicrographs of Immunohistochemistry Prepared Tissue Using ImageJ. J Vis Exp. 2018: (136): 57648.

[69] Cai J, Zhang J, Tian Y, Zhang L, Hatzakis E, Krausz KW (2017). Orthogonal Comparison of GC-MS and 1H NMR Spectroscopy for Short Chain Fatty Acid Quantitation. Anal Chem.

[70] Zhang S, Wang H, Zhu M-J (2019). A sensitive GC/MS detection method for analyzing microbial metabolites short chain fatty acids in fecal and serum samples. Talanta.

