# Supplementary material for: Reconstituting gut microbiota-colonocyte interactions reverses diet-induced cognitive deficits: The beneficial of eucommiae cortex polysaccharides
Source: Theranostics. 2024 Aug 1;14(12):4622–42. doi: 10.7150/thno.99468 (PMC11373620; doi:10.7150/thno.99468)
Supplement: Supplementary file 1 — Supplementary experimental section, figures and table. [file thnov14p4622s1.pdf]

Supporting Information

Experimental Section

1. Colonic tissue RNA extraction and RT-qPCR

In brief, total RNA was extracted from colonic tissue using 1 ml of TRIzol reagent (Invitrogen, USA). cDNA was synthesized from a total of 2 μg RNA using M-MLV reverse transcriptase (TianGen, KR116-02), and 1 μg cDNA was amplified. Real-time PCR was performed with the NovoStart SYBR qPCR SuperMix Plus kit (Novo-protein, E096) using the Bio-Rad CFX96 Fast Real-Time PCR System following the manufacturer's instructions. The qPCR program consisted of one cycle of 95°C for 10 min, 40 cycles of 95°C for 20 s, and 60°C for 1 min. Gene expression levels were normalized to that of the house-keeping gene GAPDH using the $2^{-\Delta\Delta CT}$ method.

2. 16S rRNA amplicon and sequencing

V4 region of the 16S rRNA gene was amplified using primers 515F 5′-GTGCCA GCMGCCGCGGTAA-3′ and 806R 5′-GGACTACHVGGGTWTCTAAT-3′ with barcodes. Sequencing data were processed following our previous protocols with modifications [1]. In detail, the DADA2 method was adopted for sequence denoising, generating representative sequences and abundance table [2]. In addition, we further used the LULU method on the microbiome data to remove erroneous representative sequences [3]. EZBioCloud-based Naive Bayes classifier was used to assign taxonomy to the representative sequences [4]. The diversity of gut microbiota was assessed by R package microeco (version 0.12.0) [5]. The correlation between bacterial taxa and behavioral indicators and spine density was calculated by LinkET in R (https://github.com/Hy4m/linkET).

3. Colonic tissue transcriptome profiling

Approximately 50 mg of colonic tissue was used for RNA isolation. After extraction of total RNA using TRIzol reagent (Invitrogen, cat. NO 15596026), DNA digestion was performed with DNaseI [6]. To assess the quality of the extracted RNA, A260/A280 was measured using a Nanodrop™ OneC spectrophotometer and integrity was confirmed by 1.5% agarose gel electrophoresis. Qubit3.0 with QubitTM RNA Broad Range Assay Kit (Life Technologies, Q10210) was used to quantify the qualified RNAs. Following the manufacturer's instructions, 2 μg of total RNA was used for stranded RNA sequencing library preparation using the KC-DigitalTM Stranded mRNA Library Prep Kit for Illumina® (Catalog No. DR08502, Wuhan Seqhealth Co., Ltd. China), which eliminates duplication bias in the PCR and sequencing steps by incorporating a unique molecular identifier (UMI) of 8 random bases to label the pre-amplified cDNA molecules. The library products corresponding to 200-500 bps were enriched, quantified, and finally sequenced on DNBSEQ-T7 sequencer (MGI Tech Co., Ltd. China) with PE150 model.

Following the Trimmomatic filtering process [7], low-quality reads were discarded, and any adapter contamination was removed. To reduce duplication bias introduced during library preparation and sequencing, the clean reads were further processed using in-house scripts. First, reads with the same unique molecular identifier (UMI) sequences were clustered. Next, reads within each cluster were compared pairwise, and all reads with a sequence identity of 95% or greater were grouped into separate sub-clusters. Finally, once the sub-

clusters had been generated, multiple sequence alignment was performed to generate a single consensus sequence for each sub-cluster. This removed any errors or biases introduced during PCR amplification or sequencing.

The de-duplicated consensus sequences were used for standard RNA-seq analysis. They were mapped to the reference genome of mice from ftp://ftp.ensembl.org/pub/release-87/fasta/mus_musculus/dna/ using STAR software with default parameters. Reads mapped to the exon regions of each gene were counted by featureCounts (Subread-1.5.1; Bioconductor) [8], and then RPKM was calculated. We detected differentially expressed genes between the Control and HFD groups and between the HFD and EPs group with the DESeq2 (version 1.34.0) package [9], which was visualized by the EnhancedVolcano package in R (https://github.com/kevinblighe/EnhancedVolcano). Gene set enrichment analysis was performed on genes ranked by their differential expression using ClusterProfiler (version 4.2.2) [10], and the GseaVis package in R (https://github.com/junjunlab/GseaVis).

4. Colonic tissue metabolomics

The procedures of sample extraction and instrumental analysis were performed according to the previous protocol [11]. Briefly, 20 mg of colonic tissue was also extracted in a 2 mL microcentrifuge tube with 400 μL of the same ice-cold methanol. The sample was homogenised on a TissueLyzer for 5 min at 70 Hz, followed by storage at -20 °C for 1 h before centrifugation at 18000 g for 10 min. Again, 100 μL aliquots were frozen and dried. Finally, the dried colonic tissue were rehydrated in 50 μL acetonitrile-water (2:98, v/v) solution, ready for injection and instrumental analysis.

Metabolomics was performed using a Triple TOF 5600+. A Waters Acquity UPLC HSS T3 column (100Å, 1.8 μm, 2.1 mm × 100 mm) was used for the chromatographic separation. The mobile phases consisted of 0.1% formic acid in water (A) and 0.1% formic acid in acetonitrile (B) at a flow rate of 0.4 mL/min at a temperature of 40°C. A 15 min gradient was used starting with 2% B (0-1 min), 2-15% B (1-3 min), 15-50% B (3-6 min), 50-98% B (6-7.5 min), 98% B (7.5-11.5 min), 98-2% B (11.5-11.6 min) and 2% B (11.6-15 min). HESI positive ionisation was used with a sheath gas flow of 60 L/min, aux gas flow of 10 L/min, sweep gas flow of 1 L/min, spray voltage of 2.75 kV, capillary temperature of 325 °C and aux gas heater of 400 °C. The mass spectrometer was operated with a scan range of 80-1000 Da in MS1 acquisition with a mass resolution of 70000 FWHM (Full Widths at Half Height) at 200 Da, alternating with auto MS/MS acquisition with normal collision energy averaged at 10, 50 and 100 FWHM 17,500; AGC target 2e5; max IT 50 ms.

All mass spectral data were analyzed based on R (version 4.1.3). MS1 *.wiff data were converted to *.mzXML files and MS2 *.wiff data were converted to *.mgf files in ProteoWizards MS Convert. The converted files were processed in R based on TidyMass (version 1.0.4), resulting in master peak tables aligning all samples [12]. Metabolites were annotated by the HMDB database [13]. The DESeq2 package (version 1.34.0) was used to identify the differential metabolites [9]. Quantitative metabolite set enrichment analysis (qMSEA) was performed using the R package MetaboAnalystR (version 3.3.0) to determine the biological function of altered metabolites [14].

5. Butyrate and lactate measurements

First, 30 mg of colonic content was aliquoted into a 1.5 ml Eppendorf tube containing clean

zirconia beads of 1 mm diameter. For extraction, 350 μL of ice-cold internal standard ddH_2O (6,6,6-d3-hexanoic acid, 10 μg/ml) was added. To homogenize the sample, it was placed in a TissueLyzer and subjected to 30 Hz for 30 minutes. After centrifugation at 13000 g, 4°C for 30 minutes, 100 μL of the supernatant was collected and mixed with 10 μL hydrochloric acid (5M). To complete the extraction, 100 μL of anhydrous ethyl ether was added and vortexed before being placed in an ice bath for 10 minutes. Centrifugation was repeated at 13000 g for 20 minutes and the ethyl ether layer was transferred to a new microtube containing anhydrous Na_2SO_4. To complete the derivatization, 100 μL of the ethyl ether extract was accurately transferred to a gas chromatography (GC) vial, to which 5 μL of bis (trimethylsilyl) trifluoroacetamide was added and centrifuged for 5 seconds. The vial was then sealed and stored at room temperature for 8 hours. The derivatized samples were then used for analysis.

Gas chromatography/mass spectrometry (GC/MS) analysis was carried out using the GCMS-TQ8050 NX (Shimadzu Corporation, Shionogi & Co., Ltd., JPN). The injector, ion source, quadrupole, and GC/MS interface temperatures were set at 260, 230, 150, and 280 °C, respectively. The flow rate of helium carrier gas was maintained at 1 mL/min, and 1 μl of the derivatized sample was injected with a 3 min solvent delay time and a split ratio of 10:1. The initial column temperature was 40°C and was held for 2 min, then increased to 150°C at a rate of 15°C/min and held for 1 min, and eventually ramped to 300°C at the rate of 30°C/min and maintained at this temperature for 5 min. The ionization was done in an electron impact (EI) mode at 70 eV.

6. Western blot analysis

The hippocampus and colonic tissues of the mice were lysed by RIPA lysis buffer (Solarbio, Beijing, China) then centrifuged at 1000×g for 30 min. The concentration of total protein was measured by the BCA method. Equal amount of protein was resolved using 8% SDS polyacrylamide gel. Fractionated proteins were transferred onto PVDF membranes at 220 mA for 90 min, then the membrane was washed in TBST, blocked in skim milk for 90 min, and incubated overnight on a shaker at 4°C with primary antibody, including PSD95, PPAR-γ and β-actin. The membrane was again washed three times with TBST, incubated with secondary antibody (1:3000; Proteintech) for 60 min, washed three times with TBST, and Bands were visualized using the BM Chemiluminescence Western Blotting Kit (Roche Diagnostics GmbH, Mannheim, Germany). Membranes were analyzed using the ChemiDoc Touch system (Bio-Rad, Hercules, California, USA), and band intensity was quantified using Image J software.

7. ATP measurements

ATP measurement in colonic tissue was performed by using an Enhanced ATP Assay Kit (S0027, Beyotime Biotechnology, China), according to the manufacturer's instructions.

8. Pyruvate measurements

Pyruvate measurement in colonic tissue was performed by using an Pyruvate (PA) Content Assay Kit (BC2205, Solarbio, China), according to the manufacturer's instructions.

Supplementary Table 1. The primers used in this paper

Gene	Primer F	Primer R
IL-1β	TGACGGACCCCAAAAGATGA	TCTCCACAGCCACAATGAGT
IL-6	ACCGCTATGAAGTTCCTCTC	CTCTGTGAAGTCTCCTCTCC
TNF-α	AGTCCGGGCAGGTCTACTTT	GTCACTGTCCCAGCATCTTGT
GAPDH	AGGTTGTCTCCTGCGACTGCA	GTGGTCCAGGGTTTCTTACTCC

Supplementing information

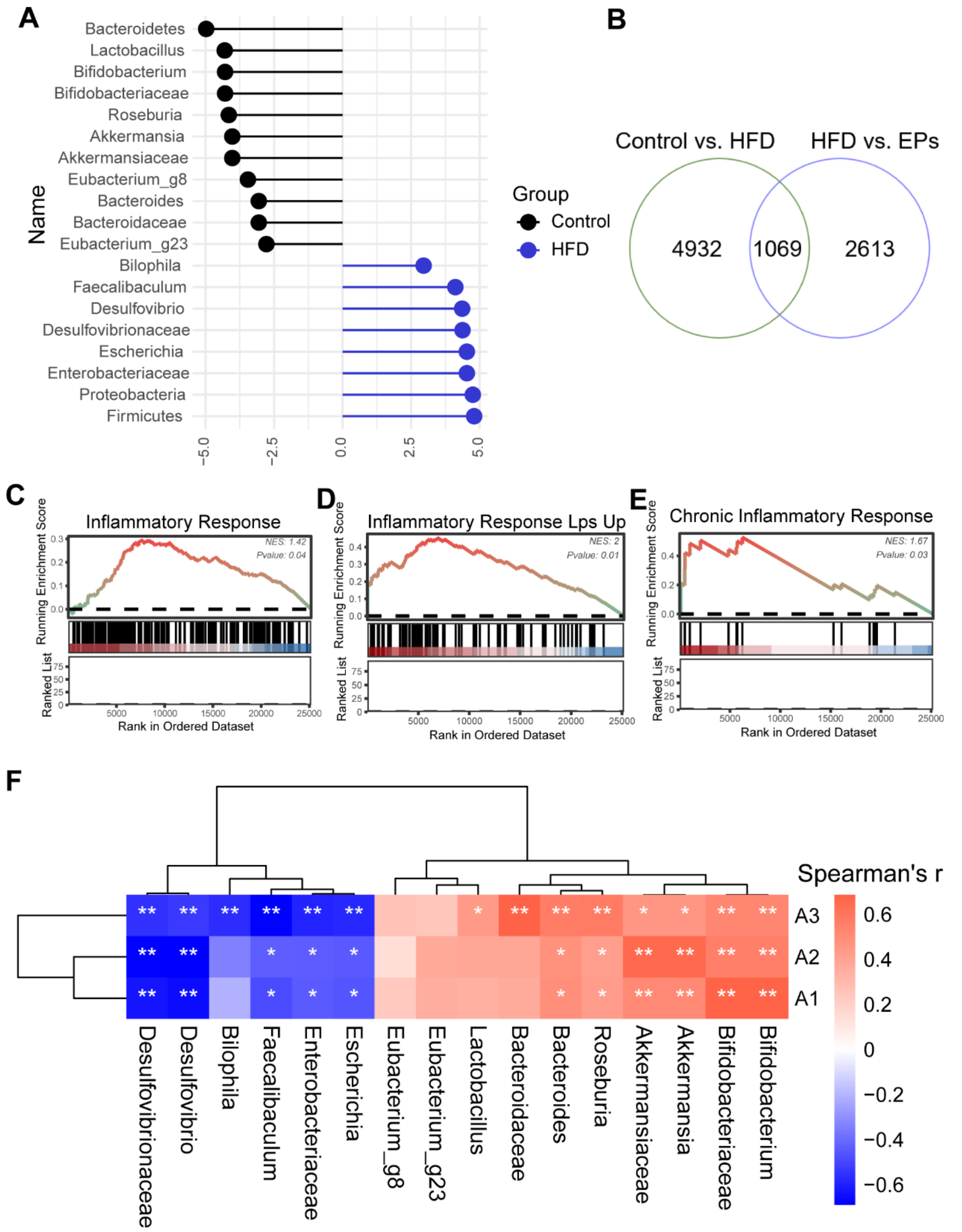

A1-Number of platform crossings
A2-Time spent in the target quadrant (%)
A3- Spine Density

Figure S1. HFD-induced gut dysbiosis was highly correlated with behavioral phenotypes of mice. (A) LEfSe analysis for differential taxa detected between Control and HFD groups (LDA value = 2.5; p-value < 0.05) (n = 8 individuals/group). (B) Venn diagram based on transcriptional profiling (n = 3 individuals/group). (C-E) Enriched gene sets of HFD−induced inflammatory response in the colon (n = 3 individuals/group). (F) Correlation between gut bacterial taxa and

behavioral indicators and dendritic spine density. *$p < 0.05$, **$p < 0.01$.

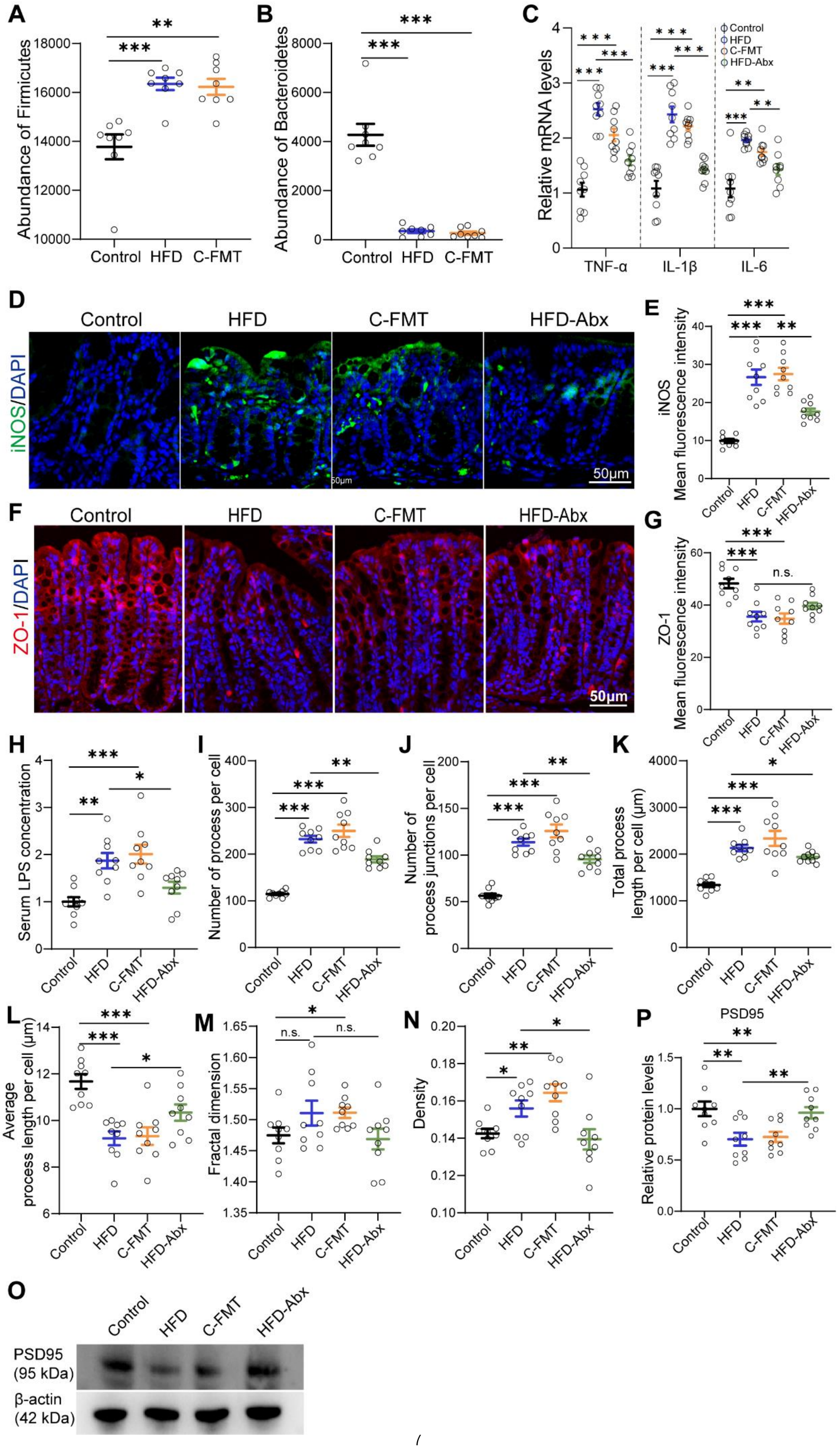
A
Abundance of Firmicutes
18000
16000
14000
12000
10000
Control HFD C-FMT
B
Abundance of Bacteroidetes
8000
6000
4000
2000
0
Control HFD C-FMT
C
Relative mRNA levels
Control
HFD
C-FMT
HFD-Abx
TNF-α
IL-1β
IL-6
D
Control
HFD
C-FMT
HFD-Abx
iNOS/DAPI
50μm
E
iNOS
Mean fluorescence intensity
F
Control
HFD
C-FMT
HFD-Abx
ZO-1/DAPI
50μm
G
ZO-1
Mean fluorescence intensity
n.s.
H
Serum LPS concentration
I
Number of process per cell
J
Number of process junctions per cell
K
Total process length per cell (μm)
L
Average process length per cell (μm)
M
Fractal dimension
n.s.
N
Density
P
PSD95
Relative protein levels
O
Control
HFD
C-FMT
HFD-Abx
PSD95 (95 kDa)
β-actin (42 kDa)

Figure S2. HFD-induced gut dysbiosis contributed to intestinal and systemic inflammation. (A, B) The abundance of the phylum *Firmicutes* and *Bacteroidetes* (n = 8 individuals/group). (C) The relative mRNA levels of cytokines (TNF-α, IL-1β and IL-6) in the colonic tissue (n = 9 for each group). (D) Representative immunohistochemical images of iNOS (green) and nuclei labeled with DAPI (blue) in the colon. (F) Representative immunohistochemical images of ZO-1 (red) and nuclei labeled with DAPI (blue) in the colon. HFD-induced gut dysbiosis increased iNOS protein expression (E) and downregulated the tight junction protein ZO-1 (G) in colonic tissue (n = 9 slices from 3 mice). (H) Serum LPS levels (n = 9 for each group). (J-N) Quantitative analysis was conducted to determine the number of processes and junctions, total and average process lengths, fractal dimensions, and morphological densities of microglia in the DG (n = 9 slices from 3 mice). (O) The expression of PSD95 in the hippocampus of different group mice was detected by western blot. The β-actin was served as the internal control. (P) Relative quantitative analysis of PSD95 protein level in the hippocampus (n = 9 for each group). Scale bars 50 μm in D and F. In C and H, data were normalized to Control. Data represent the mean ± SEM. Statistical significance was assessed using one-way ANOVA followed by Bonferroni's post hoc test. *$p < 0.05$, **$p < 0.01$, ***$p < 0.001$. Notable non-significant (and non-near significant) differences are indicated by "n.s." in the figures.

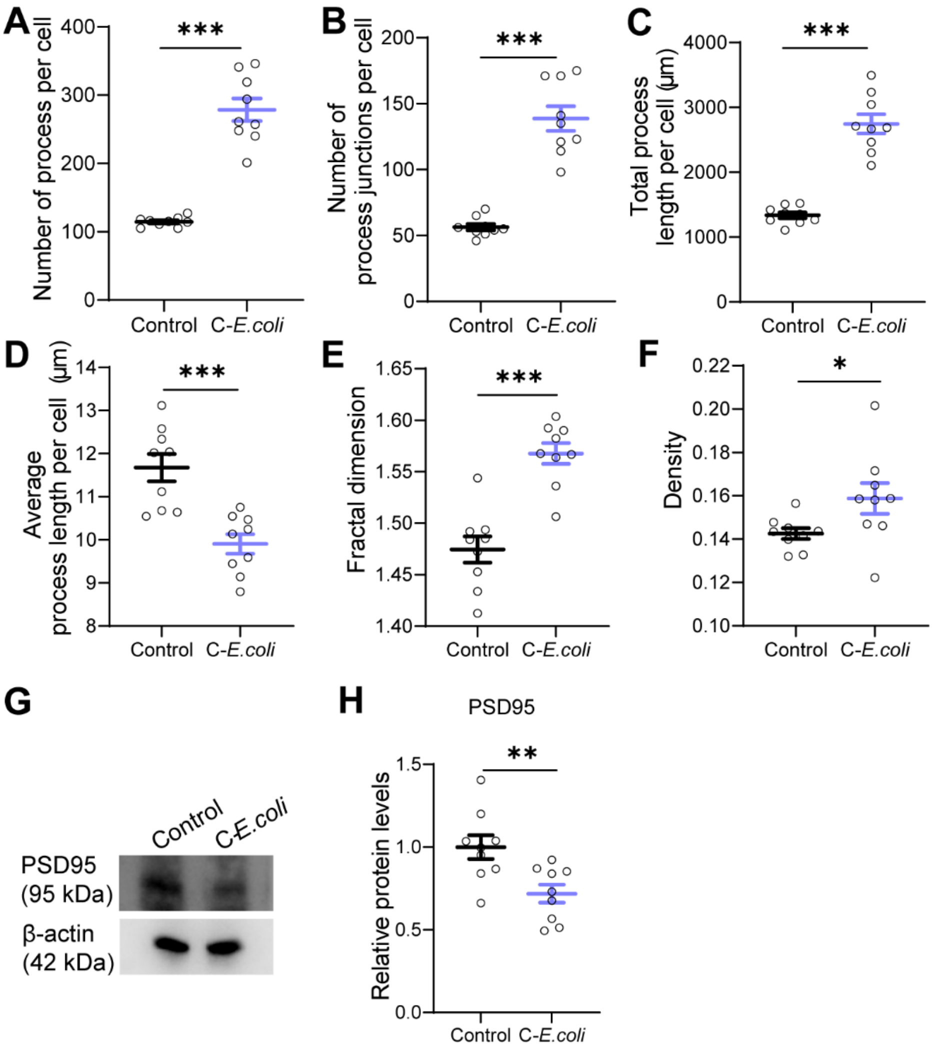

Figure S3. *E. coli* from HFD mice caused abnormal microglial morphology. (A-F) Quantitative morphological analysis was conducted to determine the number of processes and junctions, total and average process lengths, fractal dimensions, and densities of microglia in the DG of Control and C-*E. coli* mice (n = 9 slices from 3 mice). (G) The expression of PSD95 in the hippocampus of different group was detected by western blot. The β-actin was served as the internal control. (H) Relative quantitative analysis of PSD95 protein level in the hippocampus (n = 9 for each group). Data represent the mean ± SEM. Statistical significance was compared by independent samples *t*-test. *$p < 0.05$, **$p < 0.01$, ***$p < 0.001$.

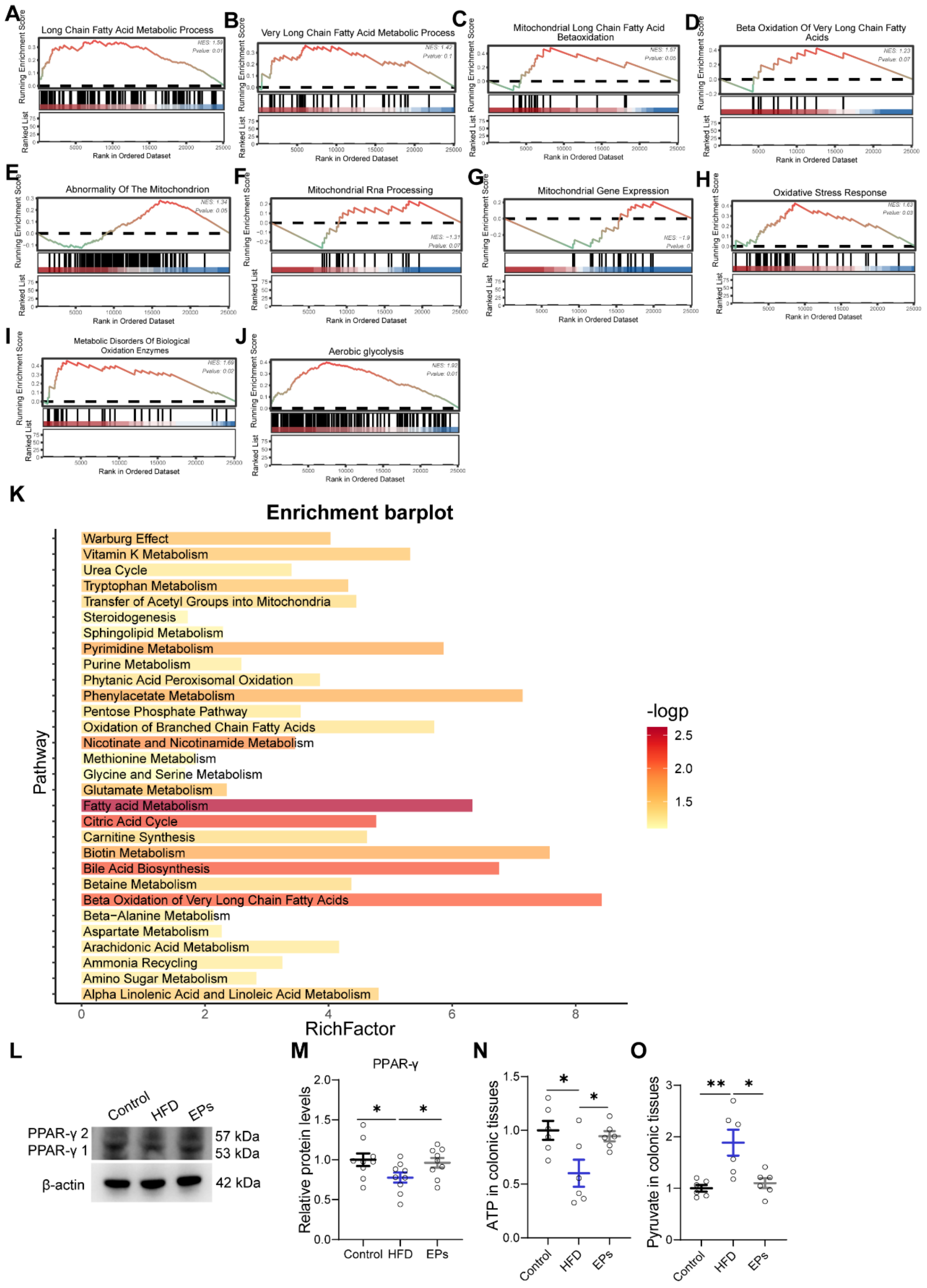

Figure S4. Sustained HFD destabilized colonic metabolic homeostasis in mice. (A-D) HFD upregulated long-chain and very long-chain fatty acid metabolic processes (n = 3 individuals/group). (E-G) HFD impaired mitochondrial function in colonocytes. (H, I) HFD triggered oxidative stress in the colon. (J) HFD induced a metabolic shift towards aerobic glycolysis in colonocytes. (K) qMSEA method was adopted to identify the colonic metabolic pathways perturbed by HFD (n = 6 individuals/group). (L) The expression of PPAR-γ in the

colonic tissues of different group mice was detected by western blot. The β-actin was served as the internal control. (M) Relative quantitative analysis of PPAR-γ protein level in the colonic tissues (n = 9 for each group). (N) Adenosine triphosphate (ATP) concentrations in colonic tissue (n = 6 individuals/group). (O) Pyruvate concentrations in colonic tissue (n = 6 individuals/group). Data represent the mean ± SEM. Statistical significance was assessed using one-way ANOVA followed by Bonferroni's post hoc test. *p < 0.05, **p < 0.01, ***p < 0.001.

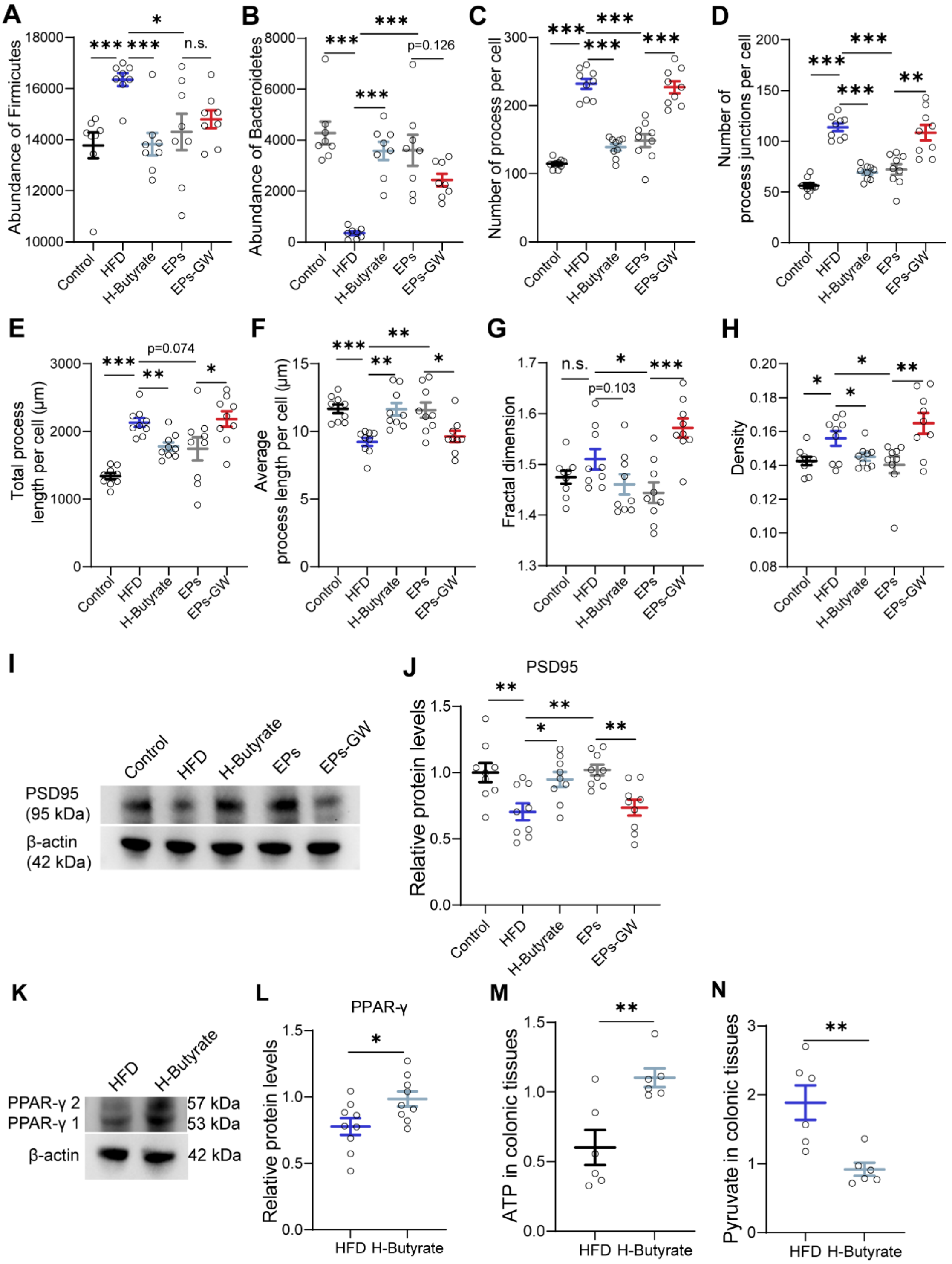

Figure S5. EPs suppressed microglia hyperactivation by remodeling gut bacteria-colonocyte interactions. (A, B) The abundance of the phylum *Firmicutes* and *Bacteroidetes* (n = 8

individuals/group). (C-H) Quantitative morphological analysis was conducted to determine the number of processes and junctions, total and average process lengths, fractal dimensions, and densities of microglia in the DG (n = 9 slices from 3 mice). (I) The expression of PSD95 in the hippocampus of different group mice was detected by western blot. The β-actin was served as the internal control. (J) Relative quantitative analysis of PSD95 protein level in the hippocampus (n = 9 for each group). (K) The expression of PPAR-γ in the colonic tissues of different group mice was detected by western blot. The β-actin was served as the internal control. (L) Relative quantitative analysis of PPAR-γ protein level in the colonic tissues (n = 9 for each group). (M) Adenosine triphosphate (ATP) concentrations in colonic tissue (n = 6 individuals/group). (N) Pyruvate concentrations in colonic tissue (n = 6 individuals/group). Data represent the mean ± SEM. Statistical significance was assessed using a one-way ANOVA followed by Bonferroni's post hoc test. *$p < 0.05$, **$p < 0.01$, ***$p < 0.001$. Notable near-significant differences ($0.05 < p < 0.1$) are indicated in the figures. Notable non-significant (and non-near significant) differences are indicated by "n.s." in the figures.

References

1. Sun P, Wang M, Li Z, Wei J, Liu F, Zheng W, et al. Eucommiae cortex polysaccharides mitigate obesogenic diet-induced cognitive and social dysfunction via modulation of gut microbiota and tryptophan metabolism. Theranostics. 2022; 12: 3637-55.
2. Callahan BJ, McMurdie PJ, Rosen MJ, Han AW, Johnson AJA, Holmes SP. DADA2: High-resolution sample inference from Illumina amplicon data. Nat Methods. 2016; 13: 581-3.
3. Frøslev TG, Kjøller R, Bruun HH, Ejrnæs R, Brunbjerg AK, Pietroni C, et al. Algorithm for post-clustering curation of DNA amplicon data yields reliable biodiversity estimates. Nat Commun. 2017; 8: 1188.
4. Zou Y, Xue W, Luo G, Deng Z, Qin P, Guo R, et al. 1,520 reference genomes from cultivated human gut bacteria enable functional microbiome analyses. Nat Biotechnol. 2019; 37: 179-85.
5. Liu C, Cui Y, Li X, Yao M. microeco: an R package for data mining in microbial community ecology. FEMS Microbiol Ecol. 2021; 97.
6. Chomczynski P, Sacchi N. The single-step method of RNA isolation by acid guanidinium thiocyanate-phenol-chloroform extraction: twenty-something years on. Nat Protoc. 2006; 1: 581-5.
7. Bolger AM, Lohse M, Usadel B. Trimmomatic: a flexible trimmer for Illumina sequence data. Bioinformatics. 2014; 30: 2114-20.
8. Liao Y, Smyth GK, Shi W. featureCounts: an efficient general purpose program for assigning sequence reads to genomic features. Bioinformatics. 2014; 30: 923-30.
9. Love MI, Huber W, Anders S. Moderated estimation of fold change and dispersion for RNA-seq data with DESeq2. Genome Biol. 2014; 15: 550.
10. Wu T, Hu E, Xu S, Chen M, Guo P, Dai Z, et al. clusterProfiler 4.0: A universal enrichment tool for interpreting omics data. Innovation (Camb). 2021; 2: 100141.
11. Lai Y, Liu C-W, Yang Y, Hsiao Y-C, Ru H, Lu K. High-coverage metabolomics uncovers microbiota-driven biochemical landscape of interorgan transport and gut-brain communication in mice. Nat Commun. 2021; 12: 6000.
12. Shen X, Yan H, Wang C, Gao P, Johnson CH, Snyder MP. TidyMass an object-oriented reproducible analysis framework for LC-MS data. Nat Commun. 2022; 13: 4365.
13. Wishart DS, Guo A, Oler E, Wang F, Anjum A, Peters H, et al. HMDB 5.0: the Human Metabolome Database for 2022. Nucleic Acids Res. 2022; 50: D622-D31.
14. Chong J, Xia J. MetaboAnalystR: an R package for flexible and reproducible analysis of metabolomics data. Bioinformatics. 2018; 34: 4313-4.
